# Impact of M36I polymorphism on the interaction of HIV-1 protease with its substrates: insights from molecular dynamics

**DOI:** 10.1186/1471-2164-15-S7-S5

**Published:** 2014-10-27

**Authors:** Mauricio GS Costa, Técio G Benetti-Barbosa, Nathan Desdouits, Arnaud Blondel, Paulo M Bisch, Pedro G Pascutti, Paulo R Batista

**Affiliations:** 1 Programa de Computação Científica, Fundação Oswaldo Cruz, Rio de Janeiro - RJ, Brazil; 2 Instituto de Biofísica Carlos Chagas Filho, Universidade Federal do Rio de Janeiro, 21949-901, Rio de Janeiro, Brazil; 3 Unité de Bioinformatique Structurale, CNRS BSPCMI UMR 3528, Institut Pasteur, 75724, Paris Cedex 14, France

## Abstract

**Background:**

Over the last decades, a vast structural knowledge has been gathered on the HIV-1 protease (PR). Noticeably, most of the studies focused the B-subtype, which has the highest prevalence in developed countries. Accordingly, currently available anti-HIV drugs target this subtype, with considerable benefits for the corresponding patients.

However, in developing countries, there is a wide variety of HIV-1 subtypes carrying PR polymorphisms related to reduced drug susceptibility. The non-active site mutation, M36I, is the most frequent polymorphism, and is considered as a non-B subtype marker.

Yet, the structural impact of this substitution on the PR structure and on the interaction with natural substrates remains poorly documented.

**Results:**

Herein, we used molecular dynamics simulations to investigate the role of this polymorphism on the interaction of PR with six of its natural cleavage-sites substrates.

Free energy analyses by MMPB/SA calculations showed an affinity decrease of M36I-PR for the majority of its substrates. The only exceptions were the RT-RH, with equivalent affinity, and the RH-IN, for which an increased affinity was found. Furthermore, molecular simulations suggest that, unlike other peptides, RH-IN induced larger structural fluctuations in the wild-type enzyme than in the M36I variant.

**Conclusions:**

With multiple approaches and analyses we identified structural and dynamical determinants associated with the changes found in the binding affinity of the M36I variant. This mutation influences the flexibility of both PR and its complexed substrate. The observed impact of M36I, suggest that combination with other non-B subtype polymorphisms, could lead to major effects on the interaction with the 12 known cleavage sites, which should impact the virion maturation.

## Background

The human immunodeficiency virus type-1 (HIV-1) has been classified in 3 groups (N, O and M). The latter accounts for 99% of the infections and is divided in nine different subtypes (A-D, F-H, J-K), more than 48 circulating recombinant forms (CRFs) and thousands of unique recombinant forms [1,2]. All approved inhibitors (targeting HIV-1 enzymes involved in key steps of viral cycle − e.g. reverse transcriptase, integrase and protease) currently in use were developed for the B-subtype (prevalent in developed countries). However, this subtype accounts only for 10% of the infections worldwide whereas non-B subtypes are prevalent in regions with the higher incidence of infections (mostly in sub-Saharan Africa) [2]. Among those targets, the protease (PR) is one of the most important in the antiretroviral therapy context. PR is responsible for the processing of Gag and Pol polyproteins, allowing virions maturation. PR inhibitors have been developed over the last 25 years, and their utilization has brought a considerable benefit for infected patients [3].

**Figure 1:**
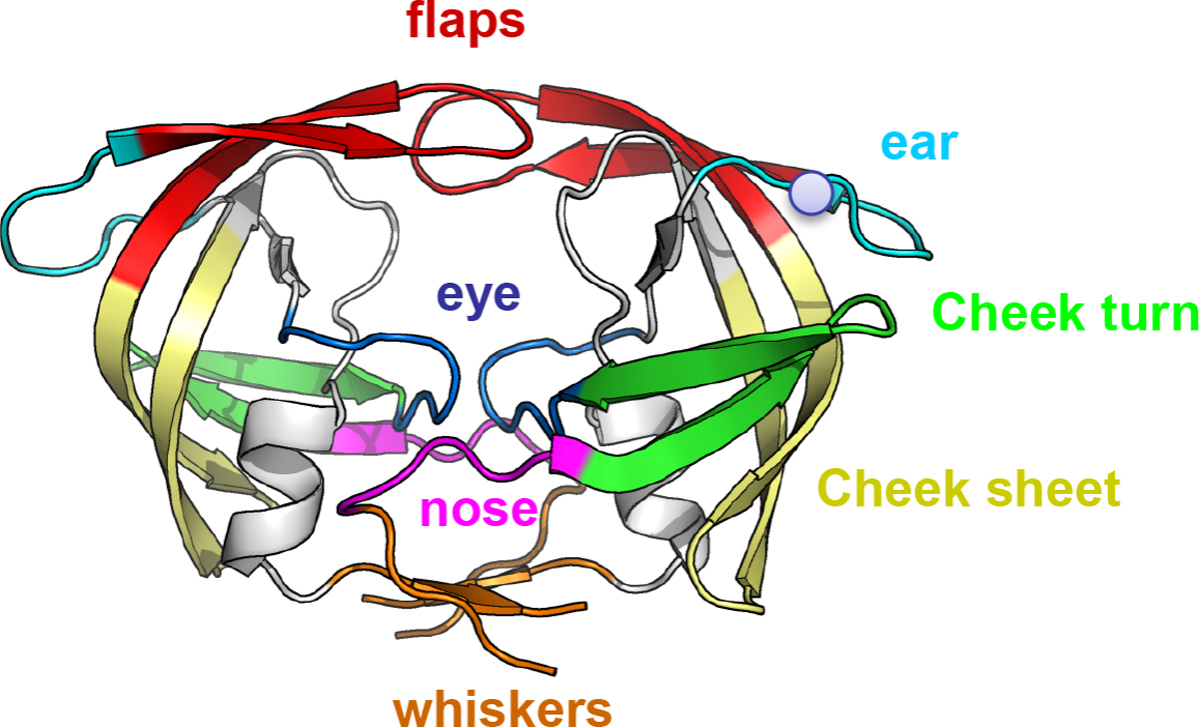
**Topology of the HIV-1 protease**. Topology of HIV-1 protease colored according to the convention proposed in Perryman et al, 2003. In red the flap domain (residues 43-58); in cyan the ears (35-42); in green the cheek turns (11-22); in yellow the cheek sheets (59-75); in blue the eyes (23-30); in magenta the nose (6-10) and in orange the whiskers (1-5 and 95-99). The 36^th ^residue is represented by a grey sphere.

There are around 450 experimentally determined available structures of this enzyme and this vast structural knowledge allows a survey of a huge number of conformations of PR complexes, with both inhibitors and substrates. Structurally, PR functions as a symmetric homodimer (99 residues each subunit), consisting in topologically different domains, as shown in Figure 1: flaps (residues 43-58); ear-flaps (35-42); cheek-turn (11-22); cheek sheet (59-75); eye (23-30 - where it is found the catalytic aspartic 25); nose (6-10) and the whiskers [4]. Structural and dynamical studies of PR normally focused on its more flexible region, the flaps, since they control the entrance/stabilization of ligands in the active site [5,6].

PR can recognize and cleave more than 12 different substrates that share no conserved motif. However PR is a symmetric dimer, this enzyme is able to recognize asymmetric substrates [7]. Crystal structures of PR complexed to six different substrates showed that their shape rather than their sequence is the main guide for the substrate recognition. The six peptides present specific hydrogen bond interactions, mainly taking place between the backbone of PR and that of the substrates [8].

Despite all the structural knowledge accumulated through the last decades, mainly for the B-subtype, there is a clear lack of information concerning interactions between non-B proteases and their ligands. Several PR polymorphisms are currently known and their effects mainly rely on reducing drug susceptibility. Among these polymorphisms, M36I is widely found in non-B proteases [9]; some authors suggest that it might be considered a genetic marker for HIV-1 group M non-B subtypes [10,11]. Although this residue is far from the PR active site, mutations in this site are often related to resistance to inhibitors such as ritonavir, nelfinavir, indinavir and atazanavir [12]. Using molecular dynamics simulations, our group has previously elucidated the molecular mechanism responsible for differences in affinity of PR from different (B and non-B) subtypes against ritonavir [13].

A previous study investigated the role of the PR M36I polymorphism on the interaction with the inhibitor nelfinavir [14]. In this paper, the authors performed 3 ns molecular dynamics (MD) simulations of PR and proposed that this mutation regulates the size of the PR binding site and thus affecting the ligand binding. Since those simulations explore a very short timescale, they would barely explore relevant conformational changes (which are frequently linked to binding cavity size regulation) [14]. Therefore, there is still a need for structural studies evaluating the impact of the PR M36I polymorphism regarding its interaction with natural substrates. Recently, based in structural analysis and computational predictions, Alvizo et al. designed a PR variant (A28S/D30F/G48R) with altered specificity for one of the substrate-cleavage sites, showing that understanding protein-protein specific contacts one is able to engineer a more stable complex [15].

Herein, we performed a set of molecular dynamics simulations (50 ns) to better understand the interactions between PR (B-subtype or M36I) and six different natural substrates. The sequences of these six substrates (Table 1) correspond to the substrate cleavage sites: *i*. within the Gag polyprotrein: matrix-capsid [MA-CA], capsid-p2 [CA-p2], p2-nucleocapsid [p2-NC] and p1-p6); and *ii*. within the Pol polyprotein: reverse transcriptase-RNaseH [RT-RH] and RNaseH-integrase [RH-IN].

**Table 1:** Sequences of the six HIV-1 PR substrates studied in this work

	Substrate	P5	P4	P3	P2	P1		P1'	P2'	P3'	P4'	P5'	PDB ID
**Gag polyprotein**	**CA-p2**	Lys	Ala	Arg	Val	Leu	*	Ala	Glu	Ala	Met	-	1F7A
	
	**MA-CA**	Val	Ser	Gln	Asn	Tyr	*	Pro	Ile	Val	Gln	-	1KJ4
	
	**p1-p6**	Arg	Pro	Gly	Asn	Phe	*	Leu	Gln	Ser	Arg	Pro	1KJF
	
	**p2-NC**	-	Ala	Thr	Ile	Met	*	Met	Gln	Arg	Gly	-	1KJ7

**Pol polyprotein**	**RH-IN**	Ile	Arg	Lys	Ile	Leu	*	Phe	Leu	Asp	Gly	Ile	1KJH
	
	**RT-RH**	-	Ala	Glu	Thr	Phe	*	Tyr	Val	Asp	Gly	Ala	1KJG

* substrate cleavage site

Binding free energies calculated from the MD trajectories with MM-PBSA revealed that for the majority of complexes, the M36I proteases have a decreased affinity against the substrates when compared to the WT (B-subtype) PR. Nonetheless, there are two exceptions: the complexes with RT-RH, with equivalent affinity, and the RH-IN substrate, with an increased affinity for the M36I PR. Essential dynamics (ED) and structural analyses allowed us to identify motions that could be related to binding affinities differences and evaluate the impact of this single polymorphism in the interaction of the PR with their substrates.

## Results and discussion

### PR complexes

From the six crystallographic structures of B-subtype PR complexed with different substrate (Table 1) available in the PDB (1F7A [7], and 1KJ4, 1KJ7, 1KJF, 1KJG, 1KJH [8]), we performed comparative modeling in order to built the M36I PR complexes, using each structure independently as template (as previously described [16] and Methods). Subsequently, solvation, ions insertion, energy minimization and consecutive MD simulations (heating, equilibration and production) were conducted for the 12 systems (6 for the B-subtype and 6 for the M36I). After an extensive equilibration, (previously described [17] and Additional file 1), we carried out production simulations with explicit solvent for 50 ns, which yielded a cumulative simulation time of 0.6 μs.

### Global structural parameters of PR

**Figure 2:**
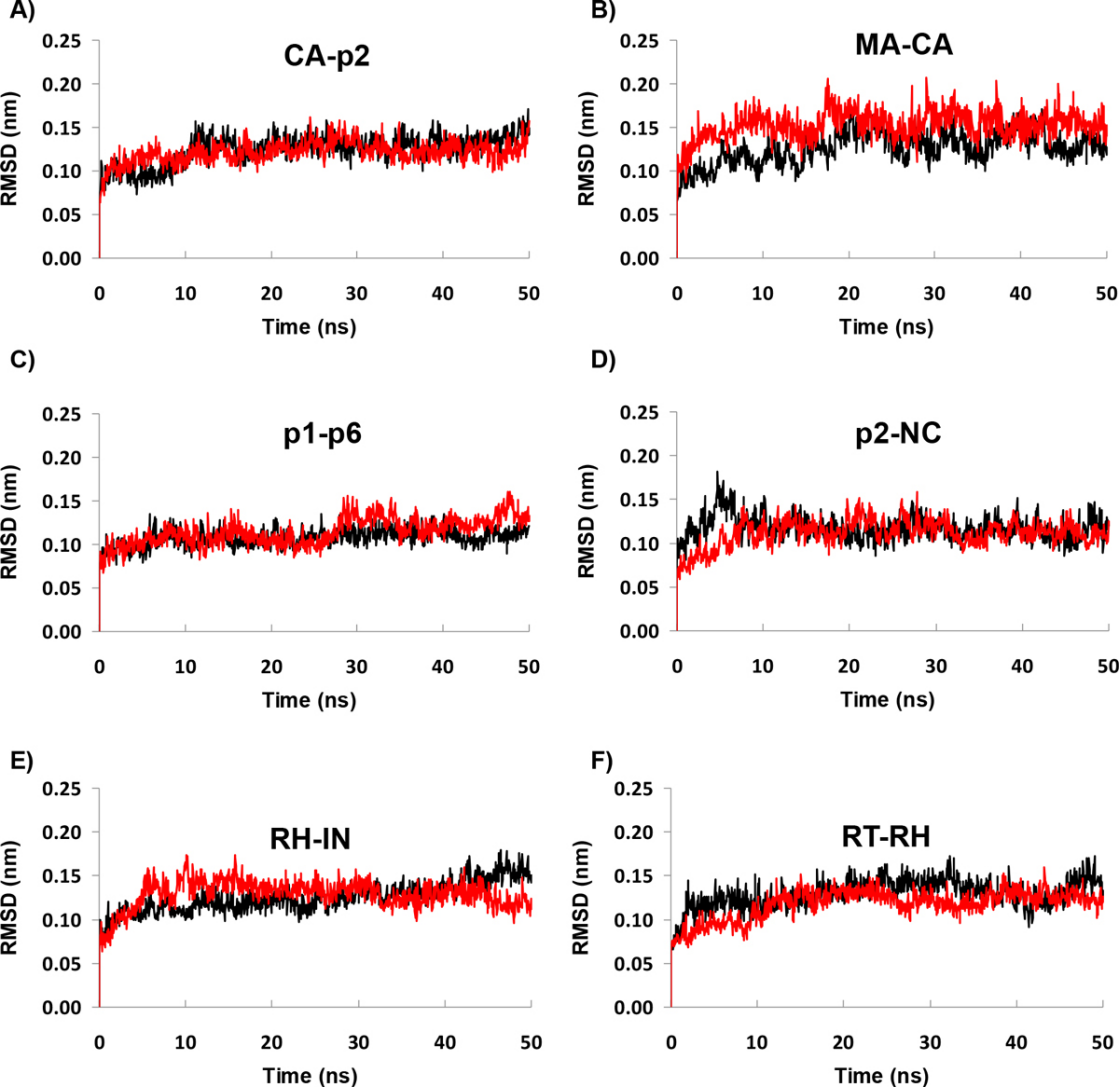
**Evaluation of the stability of trajectories:** From A to F is represented the time evolution of the RMSD of PR backbone from the initial conformation for each simulated system. The wild type enzyme (WT-PR) is represented in black and the mutant (M36I) in red.

First, we monitored the time evolution of the root mean square deviations (RMSD) of the protein backbone, as a measure of the stability of the trajectories (Figure 2). This analysis clearly revealed a similar stable behavior for all simulated systems, with deviations ranging from 0.10 to 0.15 nm. This is consistent with other studies reporting MD simulations of ligand bound forms of PR and also with the observation that generally ligand binding restricts the conformational space of proteins [5,6,16,18].

Next, to obtain further information of possible structural transitions occurred during the trajectories we performed a cluster analysis, as previously described [17]. Briefly, if during a simulation numerous clusters (based on a RMSD cut-off criteria) are visited, the system may be considered more flexible than otherwise if few clusters (densely populated) are observed. Herein each cluster contains conformations within an RMSD of 0.11 nm from its cluster center structure.

**Figure 3:**
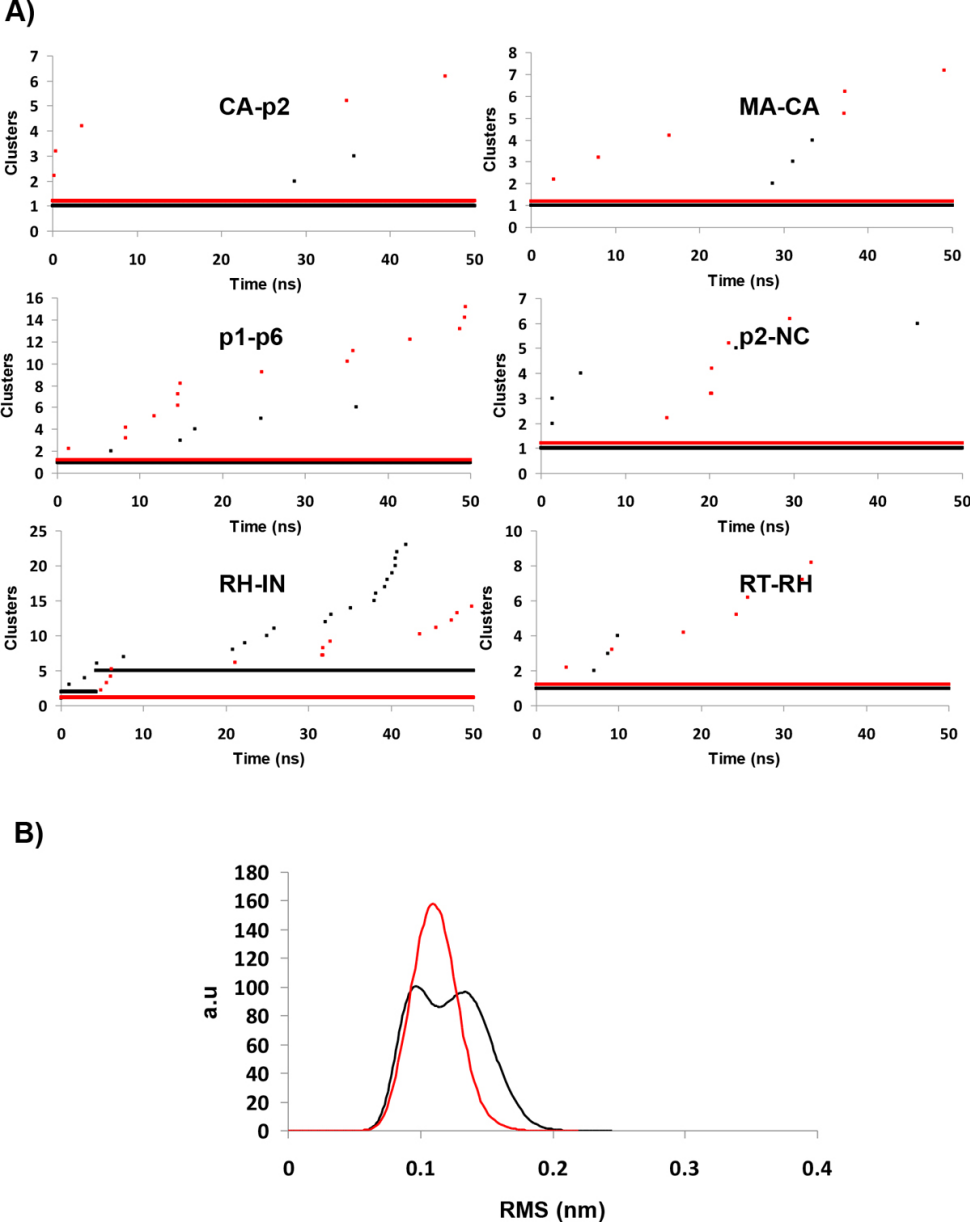
**Cluster analysis of the protease trajectories**. In A, each graph represents the time-evolution of the cluster ID assignment (PR backbone conformations) along the MD trajectories, according to the linkage clustering method (nearest neighbor), with a cut-off of 0.11 nm. In B, distribution of RMSD distances of all the pairs of conformations of the trajectory of the PR bound to the RH-IN substrate. Colored as in Fig. 2.

As displayed in Figure 3A this analysis shows that independently of the M36I polymorphism, PR stayed in the same cluster during the whole time-trajectory for all systems, except for the WT-PR - RH-IN system. In the latter, as opposed to the M36I - RH-IN system, which was stable, we clearly observed after 6 ns a shift towards a distinct PR conformation, which remained stable until the end of the 50 ns period. To confirm this result, we compared the pairwise distribution of the RMS during MD (Figure 3B). This analysis allowed us to distinguish two different populations in the PR-WT contrasting with the narrower normal distribution for the M36I PR. We also conducted the same analysis for the other simulated systems and observed a single-population distribution, independently of the presence of the M36I substitution (Additional file 2).

Next, we compared the root mean square fluctuations (RMSF) of PR backbone for each simulated system (Additional file 3). Overall, the profile and the magnitude of atomic fluctuations were similar in all simulated systems. Interestingly, after inspection of the flap hinge region, which comprises residues 34-40, for the RH-IN complexes, we noticed higher fluctuations in the WT compared to the M36I-PR; while other regions presented a similar behavior (Additional file 3).

Based on crystallographic data, Sanches *et al*. proposed that changing a long methionine residue to a shorter isoleucine (in non-B subtype PR) would lead to the adoption of a distinct conformation of the PR ear (flap hinge), which would be displaced toward the 76-83 loop [19]. This rearrangement would be responsible for a local stabilization of the flap hinge region (as shown by b-factor analysis), which would make this region more rigid than in the WT enzyme. However, according to our RMSF analysis, we only observed such a stabilization of the flap hinge on the mutant M36I-PR when it is bound to RH-IN. For the other substrates, this effect is not observed with the M36I substitution. However, this phenomenon could require the presence of other non-B subtypes polymorphism mutations to occur.

The flap region (around the Ile 50 and 149) was more flexible for some M36I PR (MA-CA, p1-p6 and p2-NC). This corresponded to a less stable behavior of these substrates during the simulations.

### Global structural parameters of the substrates

**Figure 4:**
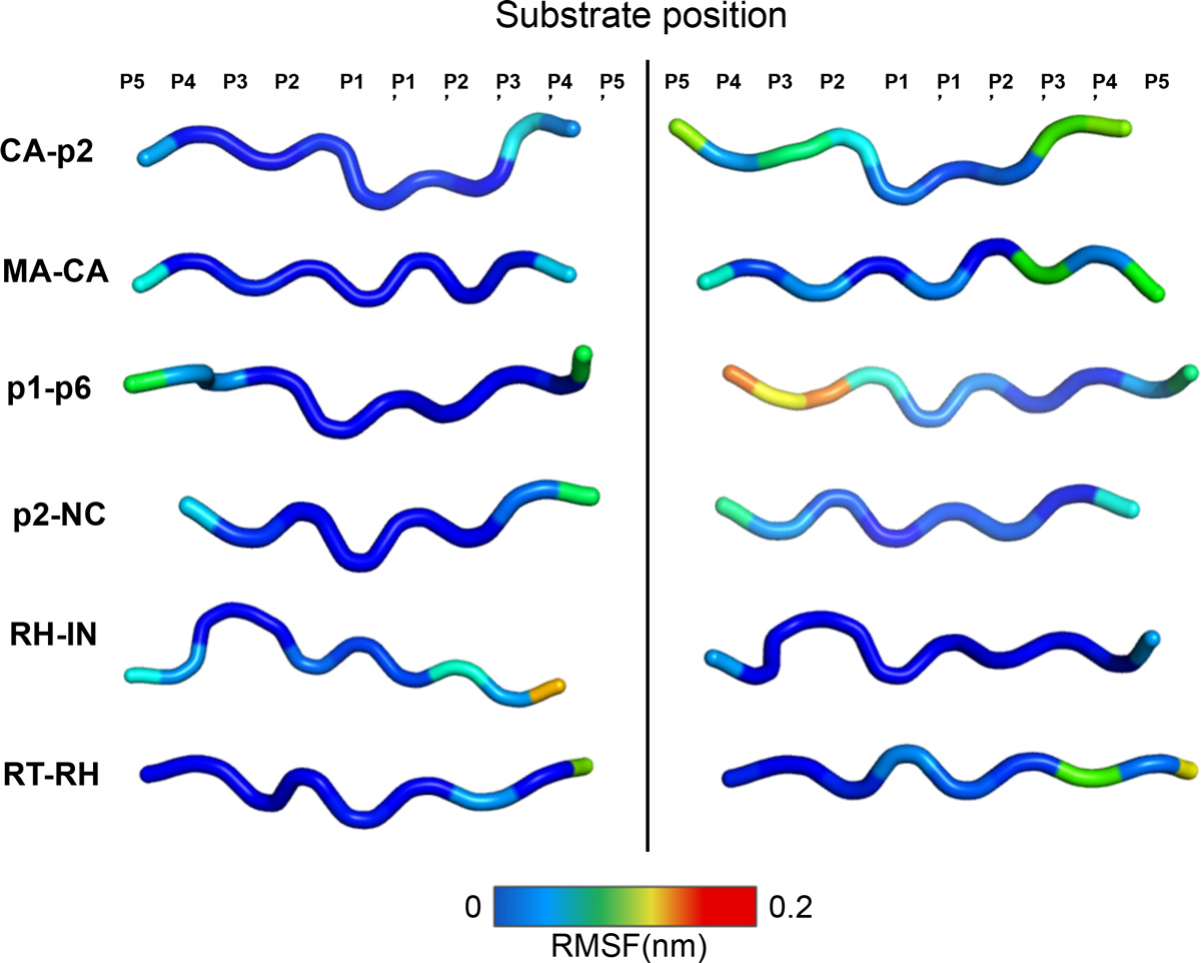
**Flexibility of the substrates' backbone**. Cartoon representation of the substrates' backbone average conformations colored according to the RMS fluctuations of each substrate residue. The substrate's groups (P5 to P5') are indicated at the top.

We compared the RMSF of the substrates' backbone during the trajectories with the same procedure as for the enzymes. To facilitate visualization of the results, we displayed the average substrate MD structures with colors indicating the RMSF of each residue (Figure 4). We observed similar profiles for each substrate bound to WT or M36I PR. For all the substrates, the central region (from P3 to P3') is very stable. The terminal groups were less stable for the M36I-complexed substrates (with the exception of the RH-IN).

**Figure 5:**
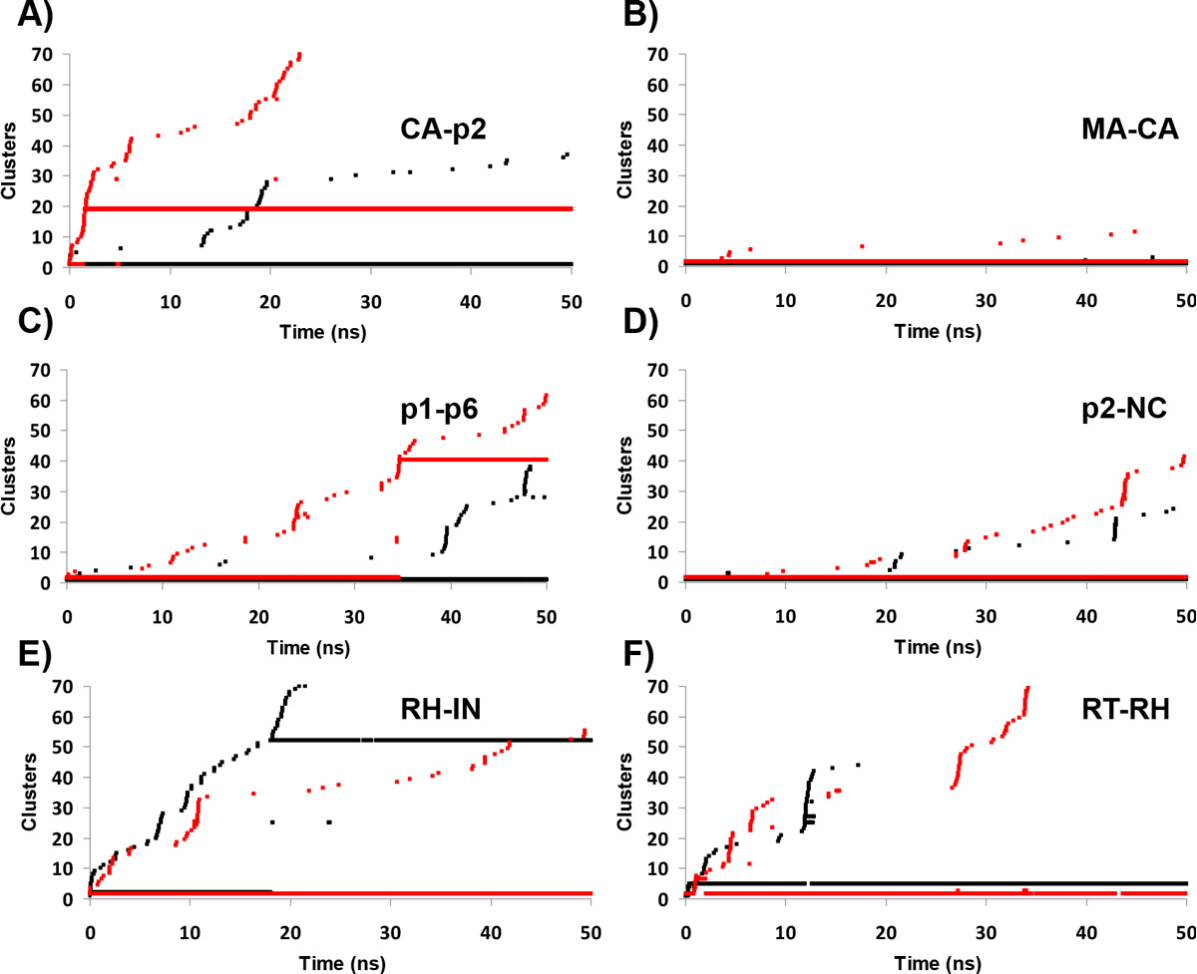
**Cluster analysis for the substrates**. From A to F the time-evolution of each cluster ID for the substrate backbone conformation along the simulations. Clustering was performed using a cut-off of 0.07 nm, with the same method as in Fig.3 (Colored as in Fig.2).

We also conducted a cluster analysis to examine the behavior of the backbone of each substrate throughout the MD trajectories. Clusters were defined here by conformations within a RMSD of 0.07 nm of its center structure. The substrates MA-CA, p2-NC and RT-RH were very stable during both WT and mutant PR trajectories (Figure 5), as also observed in Figure 4. Meanwhile, the CA-p2, p1-p6 substrates were more stable when bound to WT PR, since we observed the occurrence of a structural transition in each M36I system: around 3 ns and 35 ns, respectively. In contrast, the RH-IN substrate was more stable when bound to the mutant enzyme, which is in agreement with the RMSD and cluster distribution observed for the protein (Figures 2 and 3).

To investigate the substrate conformations sampled during the trajectories we compared the pairwise distribution of RMS (all pairs of steps in each trajectory, Additional file 4). As expected, the RMS distributions of MA-CA and p2-NC substrates presented narrower normal distributions with an average value of ~0.1 nm, in agreement with the results of the cluster analysis. Although for the RT-RH substrate, we observed broader distributions (ranging from 0.05 to 0.25 nm). It was possible to identify a very densely populated cluster centered at 0.05 nm in the WT PR simulation, contrasting with the roughly three-population distribution observed in the mutant. Based on this result, we suggest that this substrate is more stable when bound to the WT PR. Concerning the p1-p6 and RH-IN substrates, we observed one narrow distribution and one wider, bimodal one. For p1-p6 binding to the WT appeared more stable than on the mutant form. For RH-IN, by contrast, binding appeared more stable with M36I-RT.

A recent publication proposed the existence of folding preferences for the PR cleavage sites affecting kinetic parameters such as K_m _and K_cat _[20]. Using a simple regression analysis on PR/substrate crystallographic structures where the dihedral angle O (P2) - C (P2) - C (P1) - O (P1) ranges from 127.5° to 158.6°, they disclosed an inverse correlation between the magnitude of the dihedral angle and K_cat_. Considering that: *i*. a crystallographic structure is a single conformation representative of a states average; *ii*. only few complexes were evaluated and; *iii*. even in the bound state the peptide is not frozen, we decide to investigate the relevance of the assumption made in that publication. For this reason, we measured how often the dihedral angle O(P2) - C(P2) - C(P1) - O(P1) was in the 127.5° to 158.6° range. The sampling of several conformations during the trajectories presumably allows a more robust and statistically relevant analysis. We obtained frequencies below 10% for most of the substrates, except for: RT-RH (23.49% consB / 29% M36I) and MA-CA when bound to the mutant enzyme (29.78 %). The low values obtained suggest that such correlation is not likely to be taken as a good predictive factor to relate the substrates structure and kinetics.

### Essential dynamics analysis

#### Convergence and significance of the essential subspace

Several studies have demonstrated that most of the time, large amplitude motions, which are frequently implied in protein functions, involve few degrees of freedom [17,21,22]. We applied essential dynamics (ED) analysis to characterize the large amplitude motions present in the trajectories. First, it is necessary to access the quality of data, to avoid misinterpretations of the results. For that, we checked the cosine content of the first principal components (PCs), as previously described [17]. Briefly, if the cosine content is close to 1, the motions observed are likely to be representative of a random diffusion or drifting behavior. On the other hand, low values are related to correlated or equilibrated motions. We obtained very low values of cosine content for the first two PCs in all trajectories, thus indicative of genuine motions (Additional file 5).

To check the statistical significance of the motions captured by the first PCs it is important to measure the convergence of the essential subspace [23,24]. We divided the trajectories in increasing time window (t in 0 - n × 5 ns with n ranging from 1 to 10), then divided the current window in two equally sized sub-windows and performed a principal component analysis (PCA) in each one. Next, we evaluated the root mean square inner product (RMSIP) between two halves of the trajectory as previously described [17,24]. The RMSIP values were about 0.6 in all simulations and window, similar to those reported in other ED studies (Additional file 6A) [17,23,25]. Additionally, we measured the RMSIP between sequential parts of the simulation, which revealed a stable behavior during the entire simulations, thus confirming the convergence of the essential subspace (Additional file 6B).

#### Comparing the extent of sampling in WT and M36I forms

We inspected the PR conformational space described by the first two principal components, PC1 and PC2 to check whether the polymorphism affected the most relevant motions present on the trajectories. We stress here that we measured the overlaps between the eigenvectors obtained for the WT and mutant for each substrate complex considered (e.g. WT-CA-P2 vs. M36I-CA-P2) for the first two components only. In all cases, the values were extremely high ranging from 0.7 to 0.85. This was expected since the behaviors of the WT and M36I forms bound to the same substrate were similar (Figure 3 and Additional file 2). Regarding the directions of the motions, we identified high mobility mainly in the cheek-turn and ear regions (residues 11-22 and 35-42).

**Figure 6:**
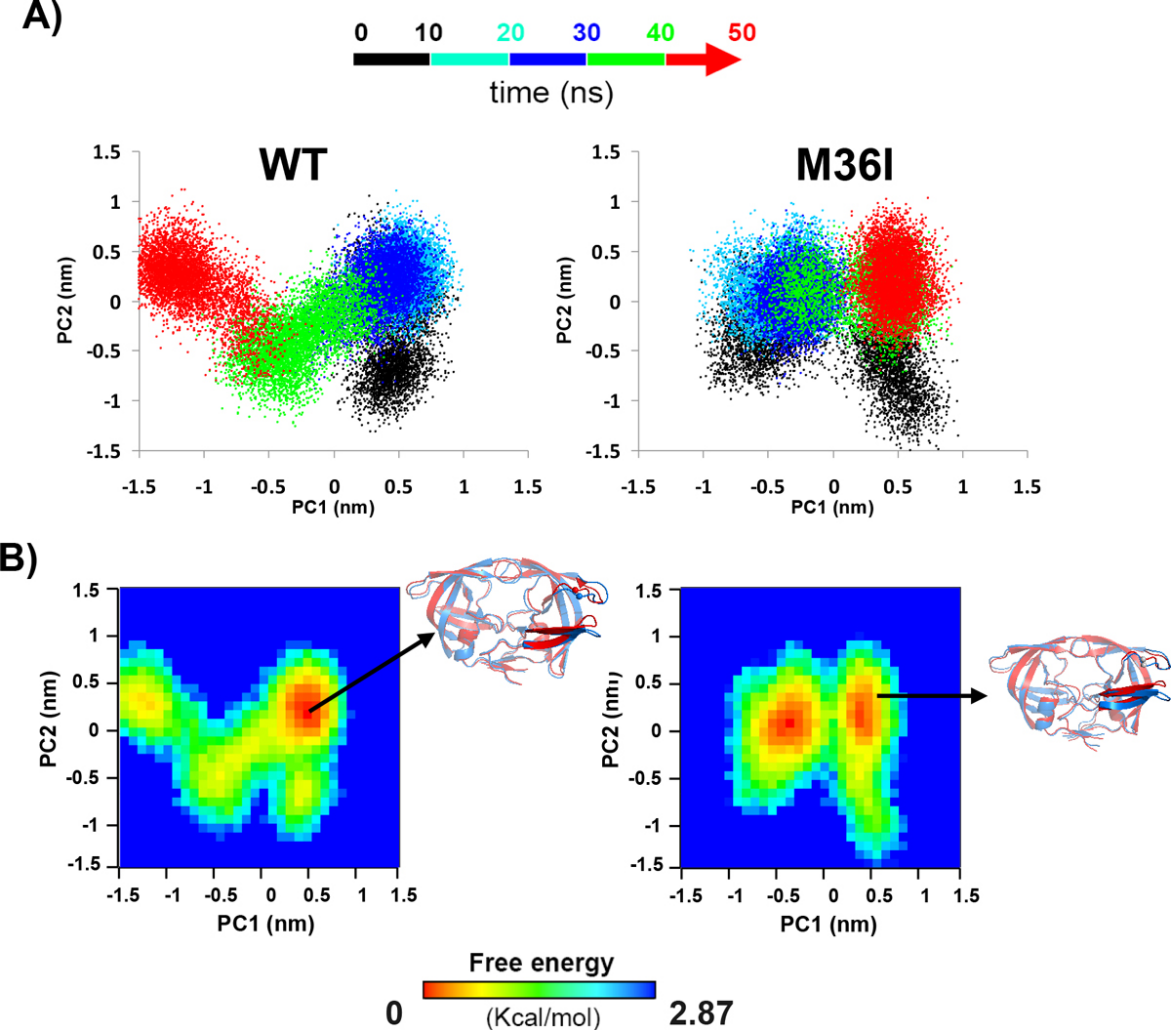
**Principal component analysis of RH-IN PR complexes**. A: The conformational sampling of WT (left) and M36I (right) PR in complex with RH-IN substrate was projected along the first two principal components fore each transient conformer. The color code represents the temporal sequence of frames (0-10 ns: black, 10-20 ns: cyan, 20-30 ns: blue, 30-40 ns: green and 40-50 ns: red). B: the free energy landscape (FEL) analysis and representative structures from the WT (left) and M36I (right) MD trajectories. The FELs were obtained using the projections of PR C-alpha atoms position vectors onto the first two principal components as reaction coordinates. Free energy values calculated from the density of distribution are given in kcal/mol as indicated by the color bar.

Additional file 7 depicts the projections of the MD trajectories of both forms onto the first two PCs. In this representation, each point is associated to each analyzed conformation of the enzyme backbone during the MD simulations; while the different colors highlight the temporal sequence of frames. Interestingly, the projection of the WT trajectories revealed a smaller extent of sampling as compared to their respective counterparts from the M36I trajectories (Additional file 7). Remarkably, the only exception was the RH-IN complexes (Figure 6A), in which the M36I form explored a smaller region than the WT one.

A free energy landscape (FEL) analysis of the WT projections revealed that the access to the lowest energy conformer at approximately 20 ns (Figure 6B). This structure resembles the starting conformation, differing solely on the ear and cheek region. Thereafter, the enzyme still explored a large portion of the conformational space, thus indicating an absence of conformational stabilization. By contrast, after oscillations during the first 10 ns, the M36I form reached a close region of the conformational space and remained there until the end of the simulation. The lowest energy conformers were accessed during the second half of the simulation (Figure 6B).

These results are indeed interesting since they demonstrate that despite the similar stable behaviors revealed by RMSD analysis, PCA projection can differentiate the WT and M36I forms in terms of the stabilization of large amplitude motions. However, it is not shown yet that the modulation of the binding affinities is due to the differences in the dynamical behavior of the WT and M36I forms.

### Understanding the structural determinants of binding affinity

Several previous studies have investigated the binding free energies of inhibitors and substrates to PR [14,16,26-29]. In general such type of analysis is performed in short trajectories (in the ps timescale), in which conformational changes rarely occur. We conducted here MM/PBSA analyses in the last nanosecond of the 50 ns trajectories, allowing the substrates to freely deviate from the starting structures and reach stable conformers. Table 2 displays the values of each contribution to the binding energy (ΔG_b_), as well as the difference between the energy obtained for the WT and mutant (ΔΔG_b_). It is important to state that we are interested in the relative energy values for each considered substrate, since the analysis of the absolute values would require a more precise free energy calculation method. Interestingly, our results were consistent with the work of Hou and collaborators in which the binding energies of WT PR and its substrates were calculated [26].

**Table 2:** Binding energies (kcal/mol) between PR and its substrates calculated with MM/PBSA.

		ΔG_vdw_	ΔG_ele_	ΔG_sol/elec_	ΔG_sol/np_	ΔG_b_^a^_total_	**ΔΔG_b_**^b^
**CA-p2**	**consB**	-75.7 ± 4	-82 ± 6	92.2 ± 12.1	-7.1 ± 0.4	-72.6 ± 2.8	**-4.1**
	**M36I**	-73.5 ± 5	-75.3 ± 5	87.6 ± 11	-7.3 ± 0.3	-68.5 ± 2.9	
**MA-CA**	**consB**	-94.7 ± 4	-66.2 ± 9	141.3 ± 14	-8.5 ± 0.4	-28.1 ± 3.4	**-7.2**
	**M36I**	-84.4 ± 7	-70.4 ± 6	142.8 ± 14	-8.9 ± 0.4	-20.9 ± 3.1	
**p1-p6**	**consB**	-85.7 ± 3	47 ± 7	-12.7 ± 9.9	-7.4 ± 0.4	-58.8 ± 1.5	**-1.6**
	**M36I**	-79.9 ± 5	37.2 ± 8	-7.7 ± 8	-6.8 ± 0.3	-57.2 ± 1.3	
**p2-NC**	**consB**	-84.9 ± 4	-51.6 ± 5	57.7 ± 12.1	-7.5 ± 0.3	-86.3 ± 2.1	**-4.8**
	**M36I**	-79.6 ± 3	-64.3 ± 5	70.4 ± 13	-8 ± 0.3	-81.5 ± 2.3	
**RH-IN**	**consB**	-74.9 ± 6	-72.4 ± 6	113.4 ± 19	-7.3 ± 0.4	-41.2 ± 2.8	**4.9**
	**M36I**	-80.5 ± 7	-77.4 ± 9	120.5 ± 17	-8.7 ± 0.3	-46.1 ± 2.6	
**RT-RH**	**consB**	-77.6 ± 6	-307.1 ± 6	356.8 ± 14	-7.3 ± 0.4	-35.2 ± 1.9	**0.6**
	**M36I**	-76.5 ± 7	-298.1 ± 12	346.1 ± 12	-7.3 ± 0.3	-35.8 ± 1.5	

^a^The entropic contribution " -*T*Δ*S*", is not included (see methods for details)

^b^Difference from the WT binding energies $\left(\text{ΔΔ}{\text{G}}_{\text{b}}=\text{Δ}{{\text{G}}_{\text{b}}}^{\text{WT}}-\text{Δ}{{\text{G}}_{\text{b}}}^{\text{M36I}}\right)$

In general, the M36I-complexes presented lower affinity than the WT for the majority of substrates, yielding negative values for ΔΔG_b_. They also presented higher flexibility in the terminal residues of the substrate during the simulations (Figure 4).

For the RT-RH substrate, M36I substitution did not change the binding affinity (ΔΔG_b _= 0.6 Kcal/mol). By contrast, RH-IN substrate, is the only one for which we clearly see an increased binding to M36I PR (ΔΔG_b _= 4.9 Kcal/mol).

Because of the sequence differences of the substrates, various reasons seem to explain changes in binding affinities. In some cases, structural modifications introduced by the mutation were sufficient to explain the results; but in most of the cases, dynamics appeared to play a decisive role. The results of each substrate will be analyzed separately for sake of clarity:

**CA-p2: **The mutated enzyme presented weaker interaction in comparison with WT probably due to the decreased contact surface between the P4, P5 and P3' groups (alanine, lysine and alanine, respectively − as shown in Additional file 8) and to the higher flexibility of these terminal groups of the substrate (Figure 4 and 5). Consequently, electrostatic interactions were weakened leading to the difference in ΔG_ele_.

**MA-CA: **Table 2 revealed stronger van der Waals interactions in the WT enzyme, which probably result from the higher stability of this form as compared with the M36I PR. Our ED analysis revealed a higher extent of sampling along the two principal components, which are related to motions on the ear to cheek region (Additional file 7). Considering that the strength of van der Waals interactions depends on the proximity between residues, the wider motions of the mutated enzyme might be the main explanation for the changes in binding affinities.

**p1-p6: **Similarly to the MA-CA complexes, the M36I PR presented more mobility along its two first principal components (Additional file 7), which may be related to the decrease in van der Waals contributions (Table 2). In addition, these motions led to the exposure and to the decrease of the contact area of the non-polar proline at P4 position of the substrate (Additional file 8), thus leading to weaker solvation energies if compared to the more stable WT.

**p2-NC: **In this system, structural and dynamical elements explain the differences in binding energies. Again, the M36I form was more mobile along the high amplitude motions described by the first PCs. This behavior led to a considerable loss of contacts from the P4 to the P2'subsite, resulting in weaker van der Waals interactions and further exposing the non-polar residue at P4 similarly as observed in the p1-p6 complex.

**RH-IN**: This system was the only for which the WT presented weaker interaction between PR and the substrate (ΔΔG_b _> 0). Here dynamics seemed to play the central role since, as previously discussed, this was the only substrate, which was more stable when bound to the mutant (Figures 4, 5 and 6). This higher stability led a stronger interaction with the residue at the P4 position (arginine), therefore increasing ΔG_ele _and ΔG_vdw _absolute contribution. In addition, the mean contact area with this substrate was higher in the M36I form, which explains the increase in ΔG_sol/np._

**RT-RH**: Here binding energies were practically the same. Accordingly our ED analysis, the mutated enzyme was more flexible than the WT (Additional file 7). However, we could observe that the conformational state reached at the end of the trajectory was similar for both systems.

### Substrate contact-area, volume and cavities calculations

**Figure 7:**
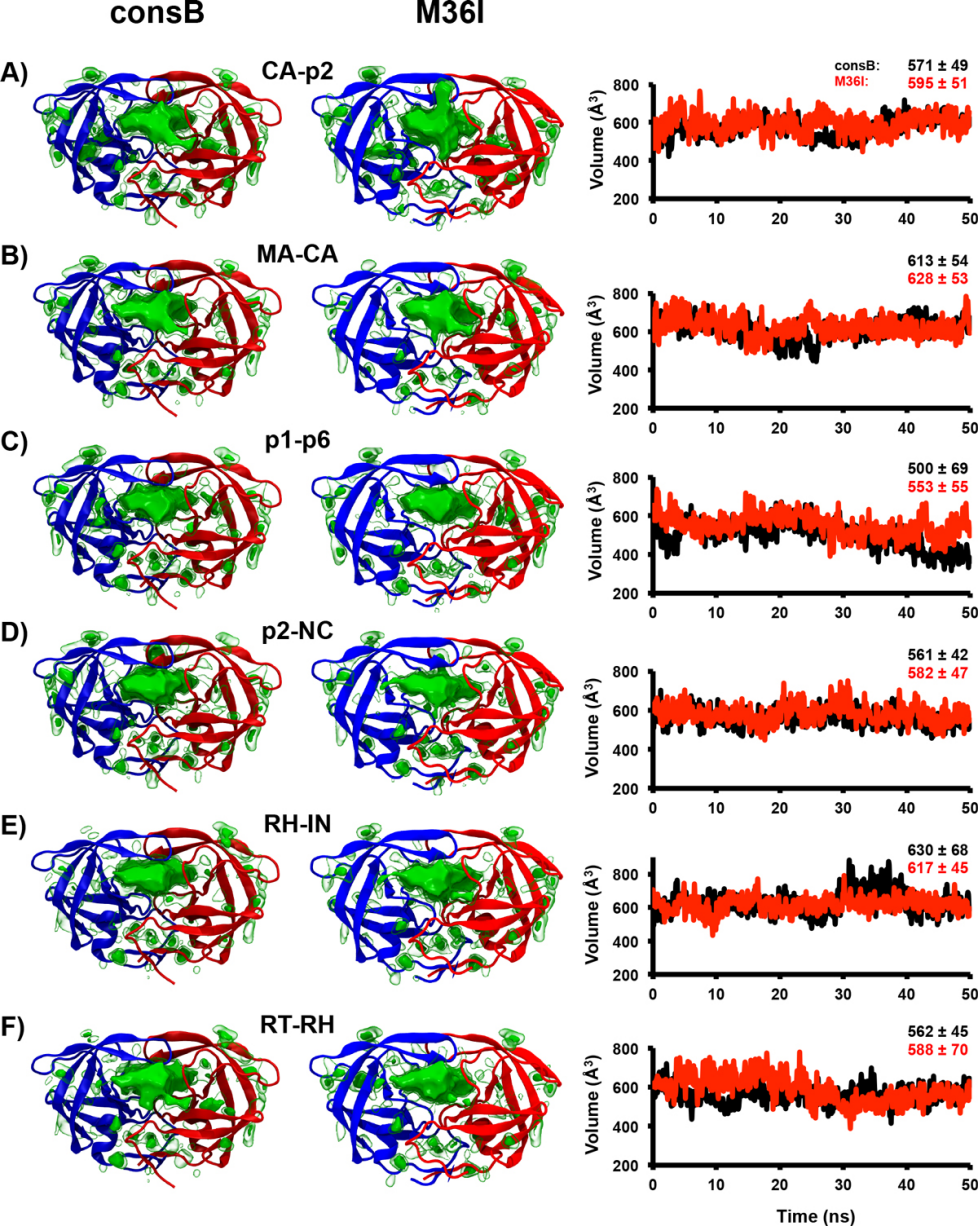
**Topology of HIV-1 protease cavities**. On the left: Time-average cavities extracted from the MD simulations represented as green surfaces. The substrates were removed and then the cavities were calculated and averaged. The biggest cavity, in all the systems, corresponded to the active site cleft. The homodimer protease was represented in cartoons colored by chain. Right: Time evolution of the volume of the main cavity (active site) during the MD simulations of the WT and M36I, in complex with the different ligands, colored as Fig.2. The average ±SD values are indicated on the top part of each plot.

Ode *et al *suggested that the role of M36I mutation was to reduce the volume of the binding cavity in the inhibitor-bound state [14]. Although large deviations were already observed in that study despite short dynamics (3 ns), we wondered if this behavior would be present in longer trajectories as we considered here (50 ns). Thus, we calculated and detected the protein cavities. Figure 7 shows the average structure and the average detected cavities in the trajectories. Then, we compared the main cavity, which corresponded to the active, for the WT and the mutant PR on all the systems by calculation of their overlap (see methods section). The overlap values range from 0.89 to 0.96, revealing very similar cavities and almost identical correspondence. We also compared the average active site of each protease (WT and mutant) in complex with the different substrates (Additional File 9).

The examination of the active site cavity volume along the MD time revealed variations during the time course of the simulations for both WT and mutant, but the average volumes observed were equivalent (Figure 7). This was in contradiction with the observations made in the previous study, and did not allow us to observe any significant variation of the binding cavity volume or surface-contact area between the substrate and PR (Additional file 8). Analysis of the contact-surface for each residue of the substrate, reveal some differences, as already discussed in the previous section, but no striking trend emerged.

## Conclusions

In this study we systematically analyzed structural and dynamical features related to the impact of the M36I mutation on the interaction of PR with six of its natural substrates. The most recognizable feature related to changes in binding affinities was the increased mobility of the ear to cheek region, as revealed by essential dynamics analyses and MMPB/SA calculations. They were correlated to increased mobility of the substrate peptide. By contrast, we observed that global structural parameters such as the cavity volume or the solvent accessible surface were mostly unaffected by the presence of the mutation. Noticeably, however, the presence of the M36I mutation seems to influence the interactions pattern and mobility at the peptide ends.

Considering the catalytic efficiency of proteases carrying mutations far from the active site, Velazquez-Campoy *et al *suggested that while binding of small molecule inhibitors was very sensitive to subtle changes of the enzyme geometry, linear peptides substrates were able to undergo conformational changes and adapt to modified cavities [30]. Thus, the observed binding energy differences are consistent with that hypothesis. A possible explanation for the differences observed for the RH-IN system, may be the gain of contacts between one or more subsites with the mutation. Indeed, we noticed a decrease of contact area between the CA-p2, MA-CA, p1-p6, p2-NC substrates and the mutant PR, in particular, interactions between the P3 and P4 groups, which were the most impacted by the presence of the mutation. By contrast, analysis of the RH-IN substrate showed that the interaction between the residue at the P4 and P5 positions and the M36I enzyme was considerably over that of the WT enzyme (Additional file 8). Interestingly, this analysis is in agreement with the results obtained from the binding energy analysis, since a higher contact is directly related to a gain in van der Waals contribution.

These affinity differences could influence in the long-term co-evolution of drug resistance-related mutations from both PR and substrate cleavage sites [31], since some of them impact in its dynamics. However, if one maintains the average substrate form (its envelope), that it known to guide the substrate binding/recognition, the PR will still performing its functions. In the case of inhibitors, the more specific interactions could be drastically changed, resulting in drug resistance.

## Methods

### Construction of M36I PR models

We have taken the following atomic coordinates from the Protein Data Bank (PDB) for the WT-PR in complex with the six substrates simulated in this work: CA-P2 (entry: 1F7A); MA-CA (1KJ4); p1-p6 (1KJF); p2-NC (1KJ7); RH-IN (1KJH); RT-RH (1KJG) [7,8]. To obtain the M36I PR structures in complex with each substrate, we carried out comparative modeling using each corresponding WT-PR crystallographic structures complex as a template. All models were built with Swiss-PDB-viewer and validated stereochemistry and energetically using Procheck and PROSA, as described in [13,16].

### Molecular dynamics simulations

Molecular dynamics (MD) simulations, energy minimization and trajectory analyses were carried out with the GROMACS 4 package [32,33] using the AMBER ffamber99sb force-field [34]. Explicit TIP3P [35] water molecules were used in all simulations, with a 0.1 nm layer of water molecules (approximately 18500 depending on the system) added around the solute molecules in a triclinic water box, using periodic boundary conditions. Counter ions were inserted to neutralize the system in addition to NaCl in 0.15 M final concentration (Na^+^: 54 ± 1; Cl^-^: 61 ± 1 depending on the system). LINCS [36] and SETTLE [37] were applied to constrain solute and solvent bonds, respectively. Temperature was kept at 300 Kelvin with a canonical rescaling approach [38]. The pressure was kept at 1 atm with the Berendsen approach [39]. Electrostatic interactions were calculated with the PME method [40], using non-bonded cutoffs of 1.0 nm for Coulomb and 1.2 nm for van der Waals. The MD integration time-step was 2 fs.

A three-step energy minimization protocol was used to avoid artifacts in atomic trajectories due to conversion of potential into kinetic energies: first, we applied the steepest-descent algorithm: *i*. 5000 steps with protein heavy atom positions restrained to their initial positions using a harmonic constant of 1 kJ/mol.nm in each Cartesian direction, allowing unrestrained water and hydrogen movement; and *ii*. 5000 steps with all atoms free to move; *iii*. subsequently, the conjugated-gradient algorithm was applied for further minimize the energy until it reached a gradient of 42 kJ/mol.nm.

Next, we performed a heating procedure from 20 to 300 K (Additional file 1A). For this purpose we performed a 500 ps MD using a "reverse" simulated annealing procedure keeping the protein heavy atoms restrained to their initial positions (using the same harmonic restraint potential as above). So the velocities were initiated at an initial temperature of 20 K and we used the "annealing" option on the ".mdp" gromacs file to heat gradually the system until reach the requested temperature (300 K), in contrast to the conventional cooling protocol, as described in [17].

Subsequently, we performed an equilibration consisting in a preliminary MD (1.0 ns), gradually reducing the positional restraint potential from 50 to 0 kJ/mol.nm with successive MD simualtions (100 ps) with restraints set to 50, 25, 10, 5, 1, 0.5, 0.1 and 0.05 kJ/mol.nm; followed by a 200 ps MD simulation with no position restraint (Additional file 1B). This procedure allows solvent and protein equilibration and avoids artifacts, as described in [17].

### Clustering analysis

We applied the g_cluster module of GROMACS package to calculate the RMS clusters of PR backbone conformations, using the method of simple linkage (nearest neighbor) with a RMSD cut-off of 0.11 nm. For the substrates, we used an RMSD cut-off of 0.07 nm.

### Cavity detections, volume and contact area calculation

Cavities were detected using an in-house software based on Lee and Richards solvent accessible surface detection algorithm [41]. Briefly, the space is divided in 0.5 Å edge length cells. Grid points accessible to a 1.4 Å radius sphere that does not overlap any protein atom are considered as void and set to 1. Grid points that are similarly accessible to a sphere of 10 Å not overlapping any protein atom are considered as bulk solvent and thus discarded (eventually set back to 0). A set of connected void grid points forms a cavity.

The volume of a cavity is approximated by multiplying the number of grid points defining it by the volume of a grid cell, which here is 0.125 Å^3^_._

Conformations of each trajectory are aligned on the same reference structure using a least-square fit algorithm. Cavities are then detected for all the conformations (every 50 ps) after depletion of the peptide. The cavity of the catalytic site always corresponded to the biggest cavity of each conformation, and was kept for analysis. The average value of each grid point is then calculated along the trajectory, which defines the average cavity distribution *D^i ^*of the trajectory *i*.

The average cavity distributions of two different trajectories *i *and *j *are compared with their overlap *O_ij_*, calculated as the Euclidean inner product of the distributions *D^i ^*and *D^j ^*divided by the geometrical mean of their Euclidean norms (k is an index running over all defined grid points):

$$O_{ij}=\frac{\sum_{k}D_{k}^{j}.D_{k}^{j}}{\sqrt{\left||D^{i}||||D^{j}|\right|}}$$

Values of *O_ij _*range from 0 (totally different distributions) to 1 (identical cavity distributions).

To calculate the contact area between the substrates and PR, we employed the SURFINMD [13,42] based on Connolly's algorithm [43].

### MM/PBSA calculations

The binding free energy was calculated using the MM/PBSA approach [44-48] in which ΔG is obtained according to Eq.1:

(1) $$\text{Δ}{\text{G}}_{\text{b}}=\text{Δ}{\text{E}}_{\text{MM}}+\text{Δ}{\text{G}}_{\text{sol}}-\text{T}\text{Δ}\text{S}$$

Here, ΔG_b _is the binding free energy in solution which is composed by the molecular mechanics (MM) interaction energy (ΔE_MM_), the solvation free energy (ΔG_sol_) and the conformational entropy contribution to binding (-TΔS). ΔG_MM _corresponds to the sum of electrostatic (ΔE_elec_) and van der Waals (ΔE_vdw_) interaction energies between protein and ligand as follows (Eq. 2):

(2) $$\text{Δ}{\text{E}}_{\text{MM}}=\text{Δ}{\text{E}}_{\text{elec}}+\text{Δ}{\text{E}}_{\text{vdw}}$$

The solvation free energy contribution can be decomposed in two parts, the electrostatic (ΔG_sol/elec_) and nonpolar (ΔG_sol/np_) terms. (3)

(3) $$\text{Δ}{\text{G}}_{\text{sol}}=\text{Δ}{\text{G}}_{\text{sol}/\text{elec}}+\text{Δ}{\text{G}}_{\text{sol}/\text{np}}$$

The electrostatic term is calculated with the APBS software [49], which solves the Poisson-Boltzmann numerically and calculates the electrostatic energy according to the electrostatic potential. It was given a dielectric constant of 1 for the interior of the protein. The reference system and the solvated had a solvent dielectric of 1 and 80, respectively. The electrostatic energy of the reference system was subtracted from that of the solvated system to yield the solvation energy. The nonpolar contribution of the solvation free energy is computed as a function of the solvent accessible area (SAS) [50], as follows:

(4) $$\text{Δ}{\text{G}}_{\text{sol}/\text{np}}=\gamma \left(\text{SAS}\right)+b$$

In this equation, *y=*0.00542 kcal/mol Å ^2 ^and *b*= 0.92 kcal/mol. The SAS was estimated using a 1.4 Å solvent probe radius with the *g_sas *module of GROMACS. The MM/PBSA calculations were performed in 100 snapshots collected from the last ns of each trajectory.

### Principal Component Analysis (PCA) and ED analysis

We applied the g_covar module of GROMACS package to obtain the covariance matrix of C-α atomic positions from each 50 ns PR trajectory. Rotation and translation motions were removed prior to covariance matrix calculation by least-squares superposition to the averaged-structure. Each element of the covariance matrix *C *is represented by:

(5) $$C_{ij}=\left\langle q_{i}-\left\langle q_{i}\right\rangle \right\rangle \left\langle q_{j}-\left\langle q_{j}\right\rangle \right\rangle$$

where q*_i _*and q*_j _*are the internal coordinates of atoms *i *and *j*. All analyses were performed with the g_anaeig module of GROMACS.

### Cosine content analysis of the firsts PCs

As the time series of the first few principal components of simulations of large proteins often resemble cosines, we evaluated the cosine content of the first two principal components to exclude the interpretation of the random diffuse motions [51].

The cosine content *c_i _*of the principal component *p_i _*is obtained from:

(6) $$c_{i}=\frac{2}{T}{\left(\int \text{cos}\left(i\pi t\right)p_{i}\left(t\right)dt\right)}^{2}{\left(\int p_{i}^{2}\left(t\right)dt\right)}^{-1}$$

where *T *is the total simulation time and *p_i_(t) *is the projection of the coordinates at time *t *on *p_i_*. When *c_i _*is close to 1 the large amplitude motions are not sampling the potential energy landscape at equilibrium but is in a non-equilibrated random diffusion regime. It has been demonstrated that insufficient sampling can also lead to high *c_i _*values, representative of random motions. Its analysis was carried out with the g_analyse module of GROMACS.

### Convergence of the essential subspace

We assume that the essential subspace of each system was defined by the five eigenvectors with higher eigenvalues (higher amplitudes) and the overlap between the essential subspace of two different groups was obtained from the RMSIP:

(7) $$RMSIP=\frac{1}{5}{\left(\overset{5}{\underset{i=1}{\sum }}\overset{5}{\underset{j=1}{\sum }}{\left(n_{i}\cdot v_{j}\right)}^{2}\right)}^{\frac{1}{2}}$$

where *n_i _*and *v_j _*are the eigenvector of different simulations (or subparts of the same simulation). The RMSIP measures how well the subspace defined by a giveset of modes (here we consider the five lowest-frequency PC modes) from a system (or a part of a MD trajectory) can include the motion indicated by the essential subspace from the other given system (or from other part of the same MD).

### Free energy landscape (FEL)

We applied the g_sham module of GROMACS package to calculate the bi-dimensional representation of the FEL calculating the free energy (G) according to the probability of finding the system in a particular state α:

(8) $$G_{\alpha }=-k_{B}T\text{ln}\left[\frac{P\left(q_{\alpha }\right)}{P_{max}\left(q\right)}\right]$$

where *k_B _*is the Boltzmann constant, *T *is the temperature of simulation; *P*(*q_α_*) is an estimate of the probability density function obtained from a histogram of the projections of each conformation sampled during MD onto two reaction coordinates (*q_i _*and *q_j_); P*_max_(*q*) is the probability of the most visited state. We considered as reaction coordinates the first two principal component vectors, being the bi-dimensional FEL calculated from the joint probability distributions *P(q_i_, q_j_)*.

## Competing interests

The authors declare that they have no competing interests.

## Authors' contributions

TGBB and MGSC contributed equally to this work. TGBB carried out all the simulations and performed preliminary data analysis. MGSC carried out the data analysis and drafted the manuscript. PRB designed and coordinated the study, worked on data analysis and drafted the manuscript. ND performed the cavity analysis and dynamics studies. AB participated in data analysis, interpreting the study and drafted the manuscript. PMB and PGP designed and coordinated the study. All authors read and approved the final manuscript.

## Supplementary Material

Additional file 1 **Summary of the heating and the equilibration procedures**. In A, the time evolution of the temperature during the heating (from 20 K to 300 K), with the protein heavy atoms positions restrained by a harmonic potential (top). The RMSD of protein backbone atoms during the heating procedure (bottom). In B, the RMSD of protein backbone atoms during the equilibration procedure, in which the harmonic restraint potential was gradually decreased from 50 kJ/mol.nm to 0 kJ/mol.nm. Click here for file

Additional file 2 **Distribution of pairwise RMSD of proteases**. From A to E is represented the distribution of pairwise RMSD distances for the PR in each simulated system (except RH-IN). Colored as Fig. 2. Click here for file

Additional file 3 **Flexibility of the PR residues**. From A to F the RMS fluctuations calculated for PR backbone atoms is represented. Protein residues are numbered from 1-99 for chain A and for 100-198 for chain B, colored as in Fig. 2. Click here for file

Additional file 4 **Distribution of pairwise RMSD of substrates**. Distribution of pairwise RMSD distances for the substrates backbone atoms when bound to the WT PR (black) or M36I (red). The RMSD between all the pairs of conformations recorded in each simulation were computed to graph their distribution. Click here for file

Additional file 5 **Significance of motions in the essential subspace**. Cosine content analysis for the first two principal components (PC1 and PC2), during the trajectories. In Supplementary Table 1 (top) are represented the cosine content of the first two PCs for the WT PR - substrates trajectories; in Supplementary Table 2 (bottom), those for the M36I PR. Click here for file

Additional file 6 **Convergence of the essential subspace**. In A, root mean square inner products (RMSIP) of the first five principal components obtained from the two halves of each trajectory (0-5 ns, 0-10 ns, 0-15 ns, ..., 0-50 ns). In B, RMSIP between sequential parts of the trajectories (t1 in 0-1.25 ns and t2 in 1.25-2.5 ns and then, t1 in (n-2)2.5-(n-1)2.5 ns and t2 in (n-1)2.5-n.2.5 ns, for n = 2 to 20). Values higher than 0.6 indicate satisfactory convergence of the simulations [24]. Click here for file

Additional file 7 **Conformational sampling along the first two PC space**. Conformational sampling of PR in complex with its substrates (except RH-IN as given in Fig. 6) obtained by bi-dimensional projection of the trajectories onto the first two PCs. Colored as in Fig. 6. Click here for file

Additional file 8 **Analysis of contact surface area**. From A to F, the average contact surface area between each substrate residue and PR are represented for the WT and the mutant. In G, the total contact area between the substrates and the enzyme for each substrate complex. Colored as in Fig. 2. Click here for file

Additional file 9 **Similarities of the active site cleft of PR in complex with different substrates**. Overlap of the active site cleft cavity of the enzyme in complex with different substrates was calculated for the WT RT (top); and for the M36I PR (bottom). Click here for file
