# Supplementary figures and images for: Impact of M36I polymorphism on the interaction of HIV-1 protease with its substrates: insights from molecular dynamics
Source: BMC Genomics. 2014 Oct 27;15(Suppl 7):S5. doi: 10.1186/1471-2164-15-S7-S5 (PMC4243740; doi:10.1186/1471-2164-15-S7-S5)

## A) Heating

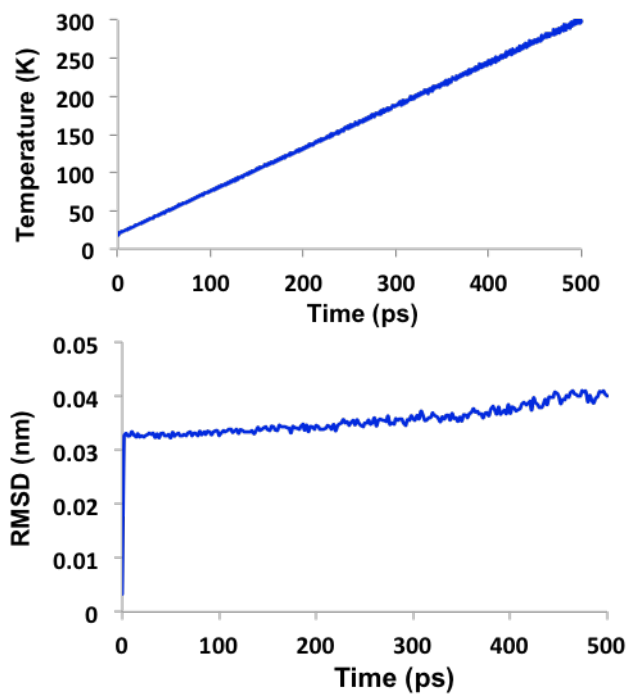

## B) Equilibration

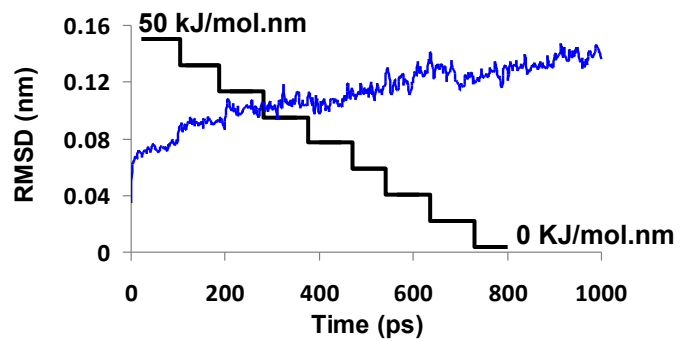

Supplement: Additional file 1 — Summary of the heating and the equilibration procedures. In A, the time evolution of the temperature during the heating (from 20 K to 300 K), with the protein heavy atoms positions restrained by a harmonic potential (top). The RMSD of protein backbone atoms during the heating procedure (bottom). In B, the RMSD of protein backbone atoms during the equilibration procedure, in which the harmonic restraint potential was gradually decreased from 50 kJ/mol.nm to 0 kJ/mol.nm. [file 1471-2164-15-S7-S5-S1.pdf]

## Additional file 2 – Distribution of pairwise RMSD of proteases

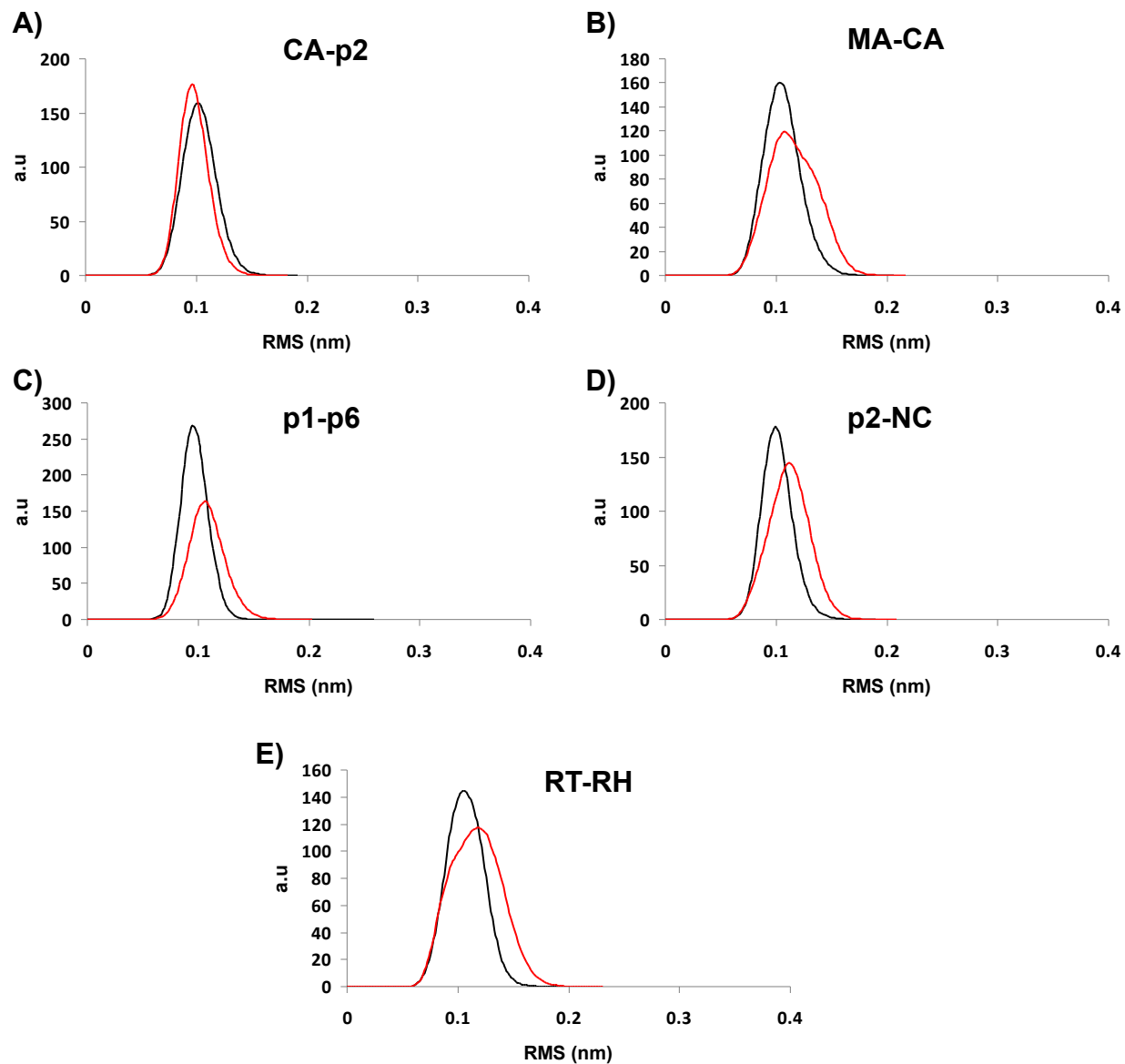

Supplement: Additional file 2 — Distribution of pairwise RMSD of proteases. From A to E is represented the distribution of pairwise RMSD distances for the PR in each simulated system (except RH-IN). Colored as Fig. 2. [file 1471-2164-15-S7-S5-S2.pdf]

### Additional file 3 - Flexibility of the PR residues

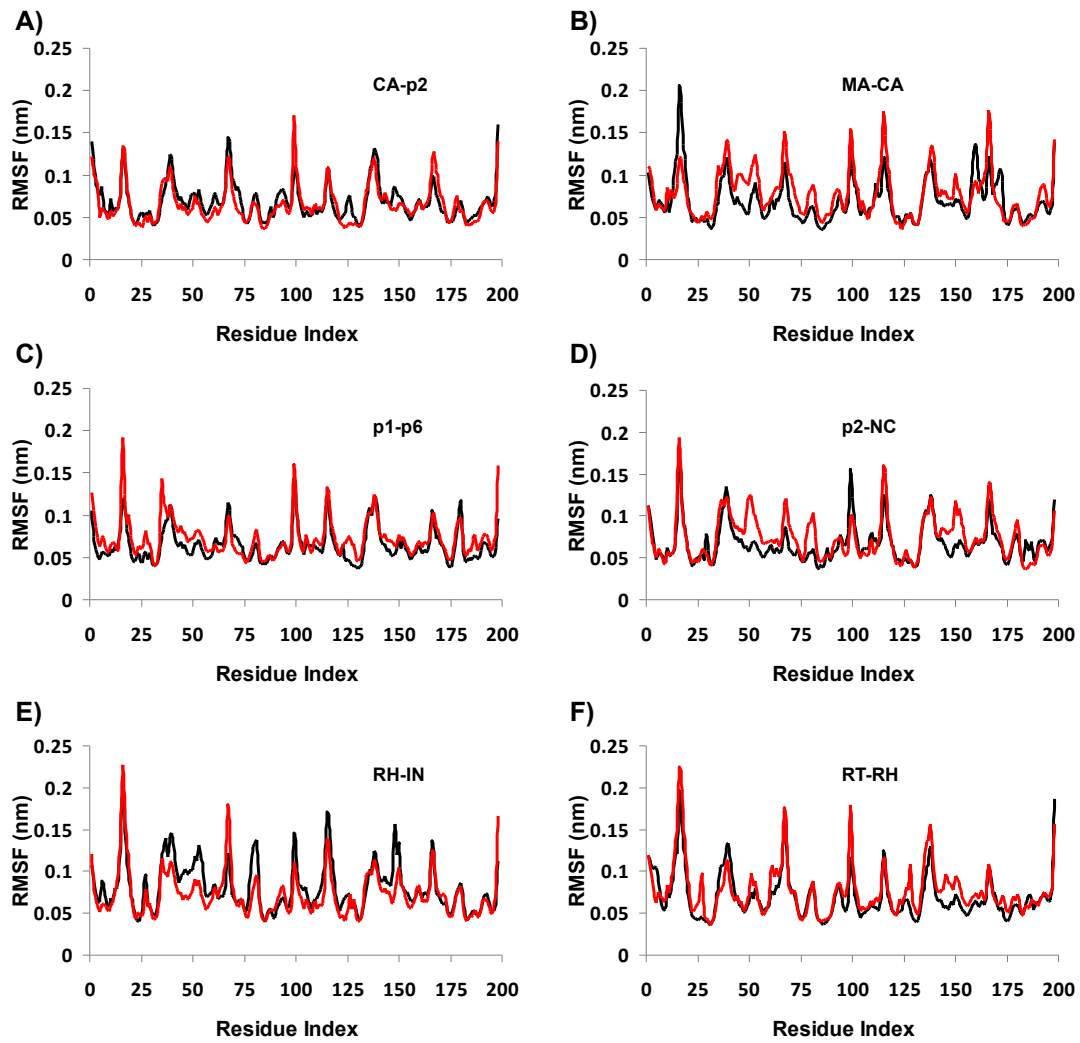

Supplement: Additional file 3 — Flexibility of the PR residues. From A to F the RMS fluctuations calculated for PR backbone atoms is represented. Protein residues are numbered from 1-99 for chain A and for 100-198 for chain B, colored as in Fig. 2. [file 1471-2164-15-S7-S5-S3.pdf]

# Additional file 4 – Distribution of pairwise RMSD of substrates

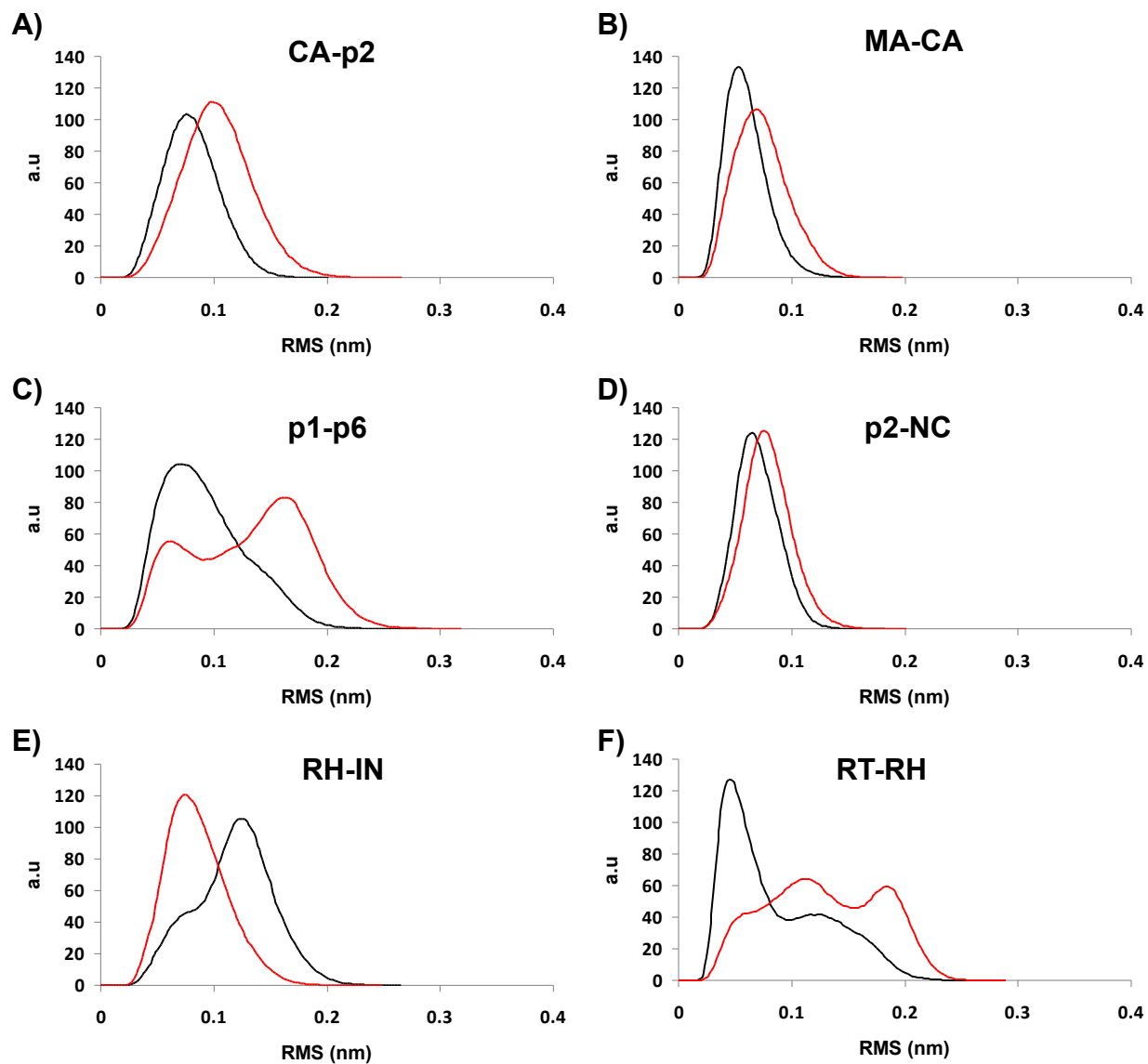

Supplement: Additional file 4 — Distribution of pairwise RMSD of substrates. Distribution of pairwise RMSD distances for the substrates backbone atoms when bound to the WT PR (black) or M36I (red). The RMSD between all the pairs of conformations recorded in each simulation were computed to graph their distribution. [file 1471-2164-15-S7-S5-S4.pdf]

Additional file 6 – Convergence of the essential subspace

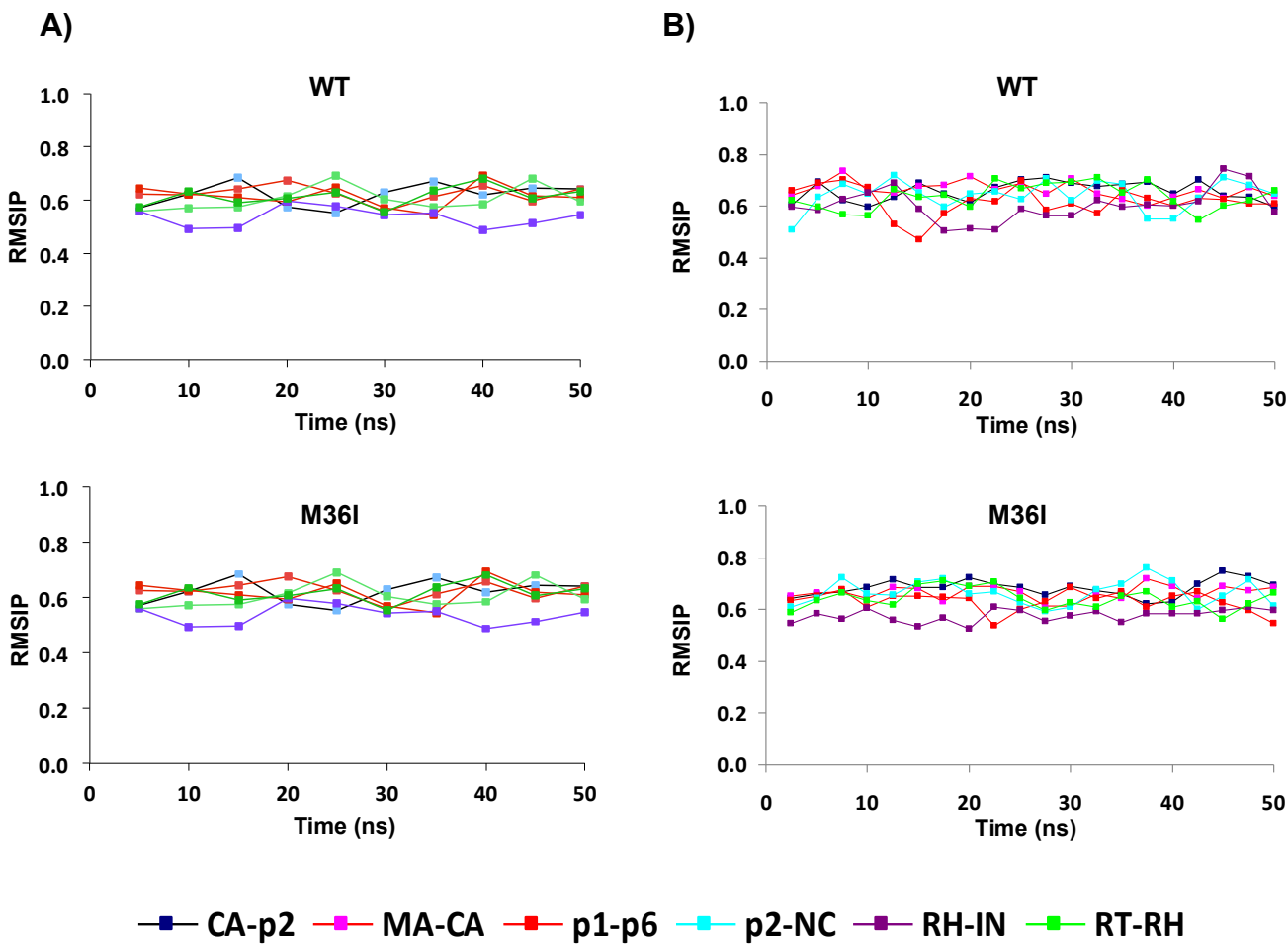

Supplement: Additional file 6 — Convergence of the essential subspace. In A, root mean square inner products (RMSIP) of the first five principal components obtained from the two halves of each trajectory (0-5 ns, 0-10 ns, 0-15 ns, ..., 0-50 ns). In B, RMSIP between sequential parts of the trajectories (t1 in 0-1.25 ns and t2 in 1.25-2.5 ns and then, t1 in (n-2)2.5-(n-1)2.5 ns and t2 in (n-1)2.5-n.2.5 ns, for n = 2 to 20). Values higher than 0.6 indicate satisfactory convergence of the simulations [24]. [file 1471-2164-15-S7-S5-S6.pdf]

Additional file 7 – Conformational sampling along the first two PC space

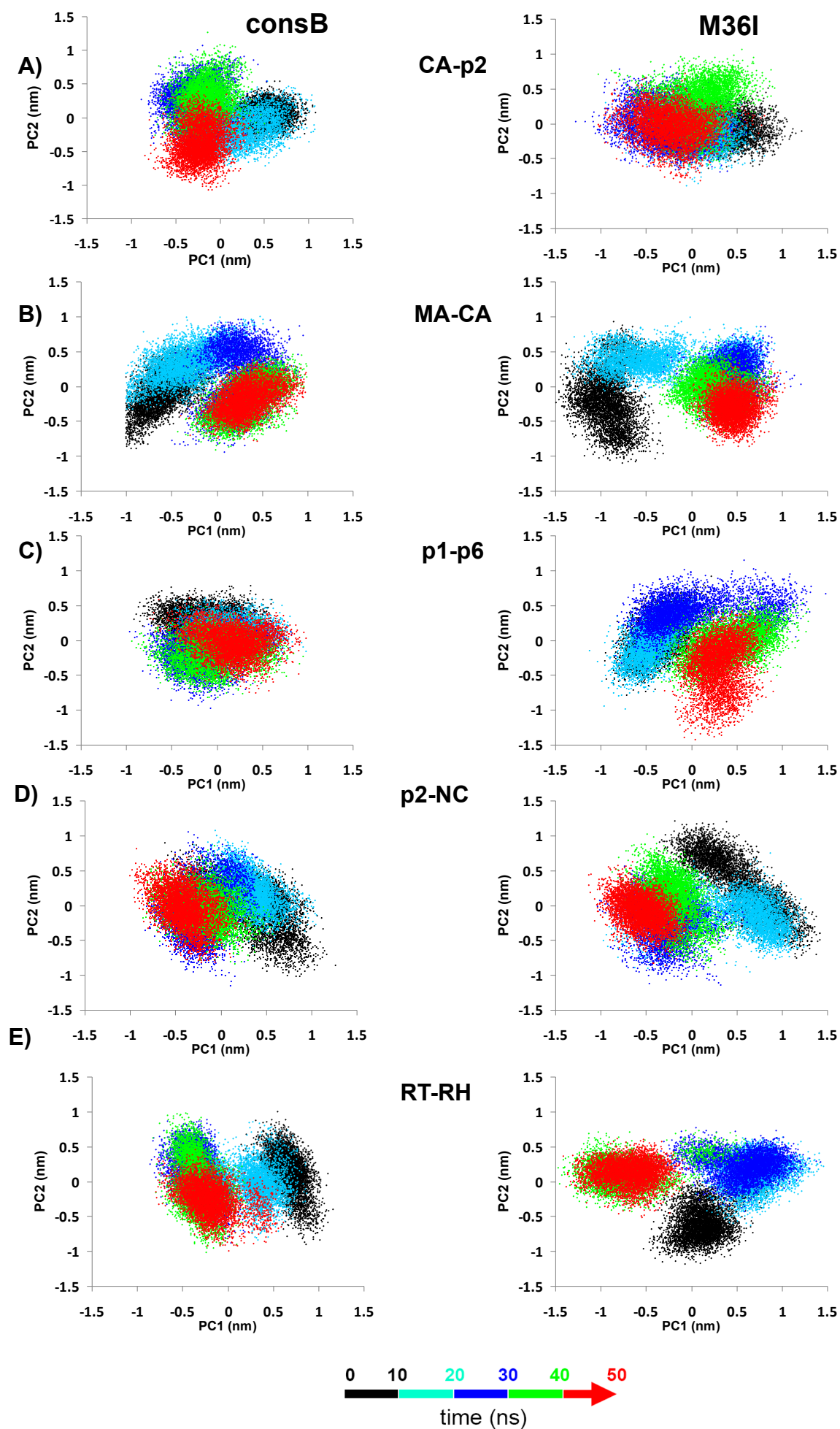

Supplement: Additional file 7 — Conformational sampling along the first two PC space. Conformational sampling of PR in complex with its substrates (except RH-IN as given in Fig. 6) obtained by bi-dimensional projection of the trajectories onto the first two PCs. Colored as in Fig. 6. [file 1471-2164-15-S7-S5-S7.pdf]

Additional file 8 – Analysis of contact surface area

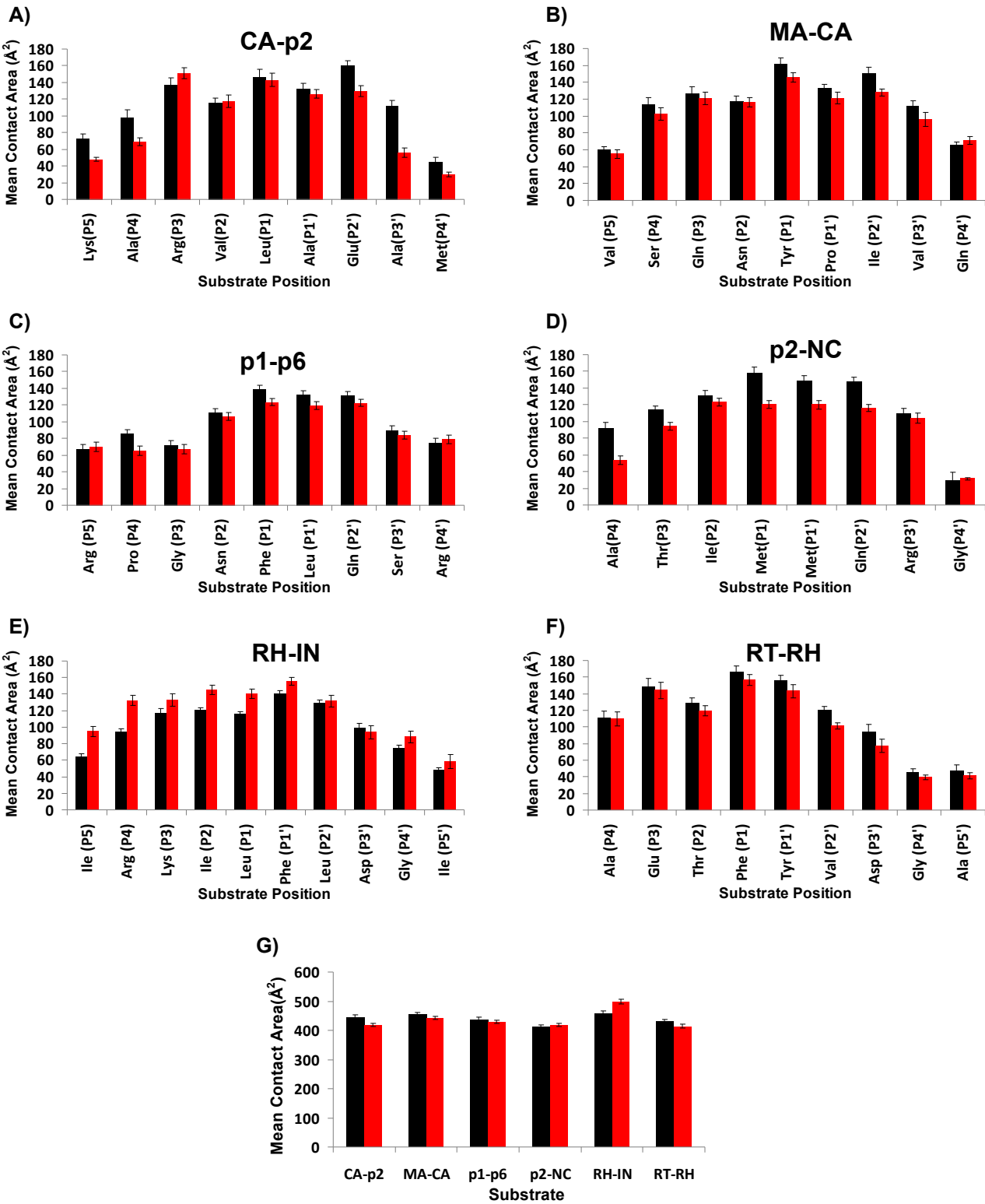

Supplement: Additional file 8 — Analysis of contact surface area. From A to F, the average contact surface area between each substrate residue and PR are represented for the WT and the mutant. In G, the total contact area between the substrates and the enzyme for each substrate complex. Colored as in Fig. 2. [file 1471-2164-15-S7-S5-S8.pdf]

Figure 9 - Similarities of the active site cleft of PR in complex with different substrates

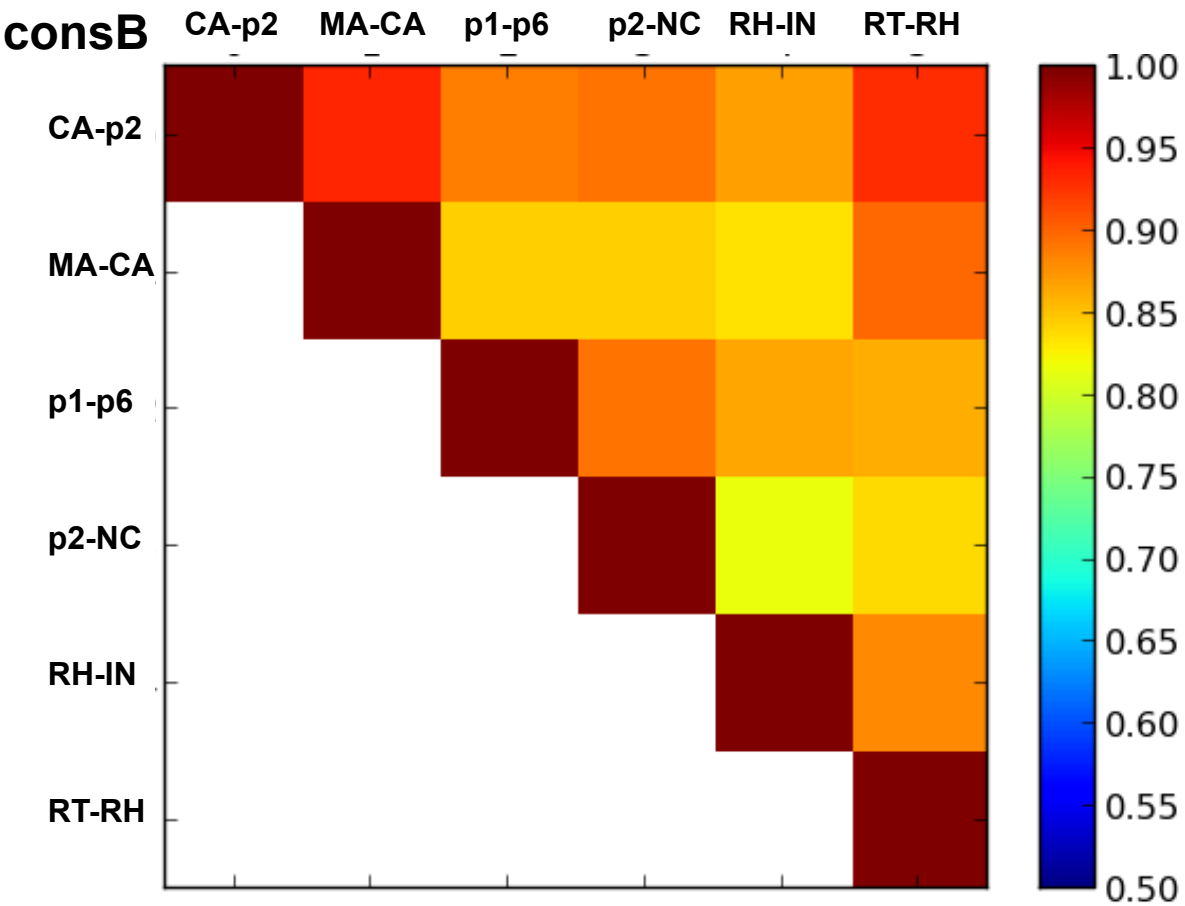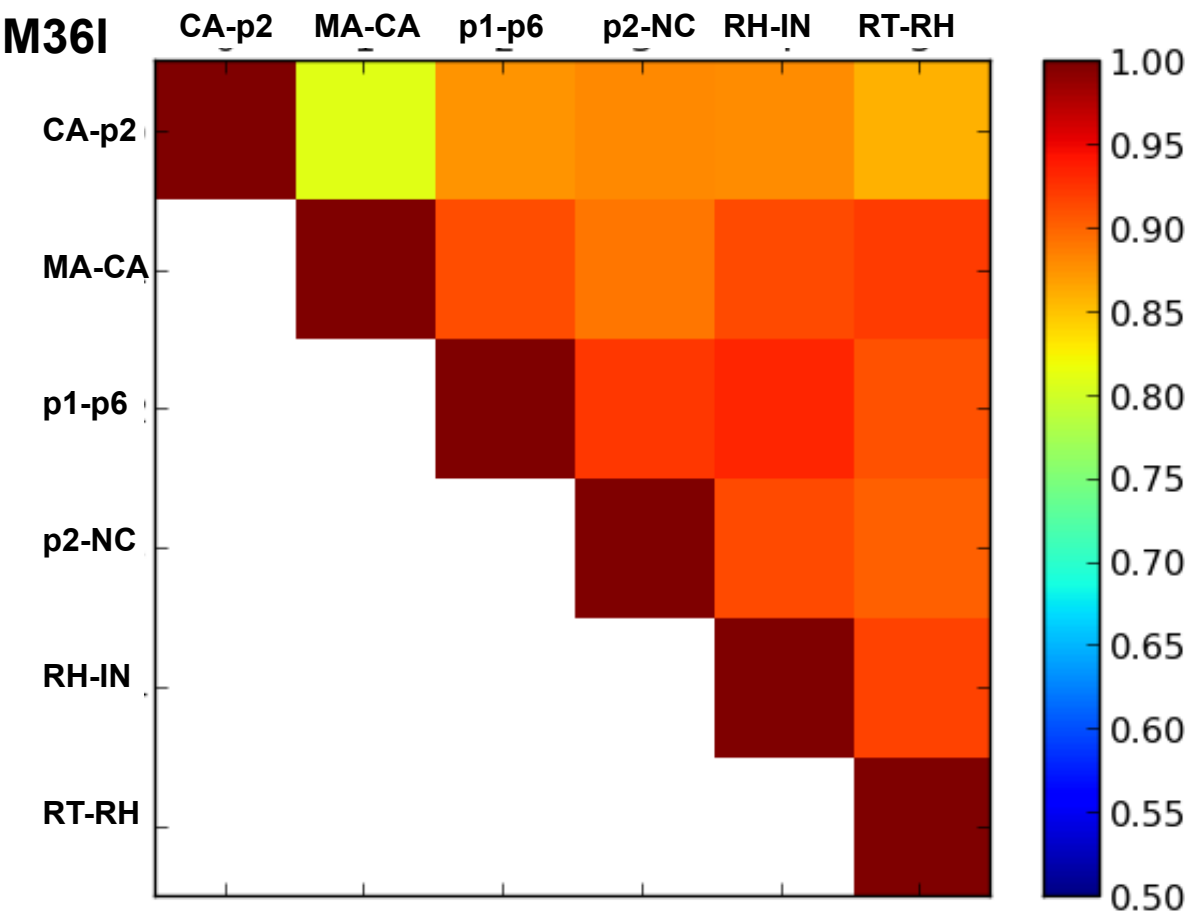

Supplement: Additional file 9 — Similarities of the active site cleft of PR in complex with different substrates. Overlap of the active site cleft cavity of the enzyme in complex with different substrates was calculated for the WT RT (top); and for the M36I PR (bottom). [file 1471-2164-15-S7-S5-S9.pdf]
