# Supplementary material for: Impact of M36I polymorphism on the interaction of HIV-1 protease with its substrates: insights from molecular dynamics
Source: BMC Genomics. 2014 Oct 27;15(Suppl 7):S5. doi: 10.1186/1471-2164-15-S7-S5 (PMC4243740; doi:10.1186/1471-2164-15-S7-S5)
Supplement: Additional file 5 — Significance of motions in the essential subspace. Cosine content analysis for the first two principal components (PC1 and PC2), during the trajectories. In Supplementary Table 1 (top) are represented the cosine content of the first two PCs for the WT PR - substrates trajectories; in Supplementary Table 2 (bottom), those for the M36I PR. [file 1471-2164-15-S7-S5-S5.pdf]

Additional file 5– Significance of motions in the essential subspace

Supplementary Table 1: Cosine content for the WT PR – substrates trajectories

**Cosine content – WT PR simulations**											
		**Trajectory time interval (ns)**									
**Substrate**	**Principal Component**	**0-5**	**5-10**	**10-15**	**15-20**	**20-25**	**25-30**	**30-35**	**35-40**	**40-45**	**45-50**
**CA-p2**	PC1	0.0052	0.0037	0.4323	0.0076	0.0056	0.0939	0.0022	0.0306	0.0014	0.0026
	PC2	0.0267	0.0672	0.0951	0.0615	0.4556	0.0526	0.0780	0.3314	0.2745	0.0359
**MA-CA**	PC1	0.0108	0.0117	0.0611	0.2941	0.0076	0.0667	0.0478	0.1528	0.0320	0.0372
	PC2	0.0473	0.0772	0.0110	0.0171	0.0182	0.0053	0.1289	0.3407	0.1036	0.0788
**p1-p6**	PC1	0.0518	0.0349	0.2422	0.0892	0.4947	0.0030	0.0843	0.3033	0.0876	0.0577
	PC2	0.0320	0.0005	0.0012	0.0300	0.2947	0.0916	0.0875	0.0362	0.2912	0.2276
**p2-NC**	PC1	0.3936	0.0009	0.0019	0.0277	0.3310	0.0623	0.0016	0.0011	0.0137	0.0627
	PC2	0.2215	0.4008	0.0555	0.0745	0.2222	0.0161	0.2627	0.1061	0.0243	0.0519
**RH-IN**	PC1	0.0076	0.0043	0.0006	0.0061	0.0010	0.1334	0.3805	0.0123	0.0337	0.0047
	PC2	0.0635	0.0066	0.0088	0.0109	0.0022	0.1380	0.0587	0.0337	0.3788	0.0076
**RT-RH**	PC1	0.0766	0.0025	0.1483	0.0872	0.0051	0.0009	0.0007	0.1770	0.0070	0.0677
	PC2	0.0004	0.0021	0.0082	0.5428	0.0352	0.0002	0.0085	0.0219	0.1791	0.0264

Supplementary Table 2: Cosine content for the M36I PR – substrates trajectories

**Cosine content – M36I PR simulations**											
		**Trajectory time interval (ns)**									
**Substrate**	**Principal Component**	**0-5**	**5-10**	**10-15**	**15-20**	**20-25**	**25-30**	**30-35**	**35-40**	**40-45**	**45-50**
**CA-p2**	PC1	0.0388	0.0376	0.3700	0.0005	0.0785	0.0001	0.0207	0.2163	0.0450	0.0705
	PC2	0.0009	0.0086	0.0204	0.1575	0.0006	0.4420	0.0052	0.4792	0.0232	0.0474
**MA-CA**	PC1	0.0043	0.0074	0.0567	0.6017	0.0867	0.0080	0.0553	0.0009	0.0196	0.0028
	PC2	0.1063	0.6795	0.0057	0.0013	0.0023	0.0041	0.1478	0.0402	0.0141	0.0046
**p1-p6**	PC1	0.0149	0.0139	0.0013	0.1068	0.0136	0.4219	0.0059	0.0358	0.0108	0.0185
	PC2	0.0736	0.1058	0.0061	0.3606	0.0049	0.0124	0.0158	0.0956	0.0908	0.0490
**p2-NC**	PC1	0.0716	0.0203	0.0017	0.0025	0.0008	0.0052	0.0003	0.0003	0.0368	0.0096
	PC2	0.0556	0.0165	0.0018	0.0017	0.0639	0.3417	0.0993	0.0122	0.0004	0.0003
**RH-IN**	PC1	0.0697	0.0113	0.0001	0.1038	0.0362	0.1564	0.5793	0.4142	0.0042	0.0003
	PC2	0.0860	0.0177	0.2785	0.0766	0.1524	0.0003	0.5031	0.2744	0.0055	0.0442
**RT-RH**	PC1	0.1436	0.1891	0.0007	0.0045	0.0001	0.1206	0.0686	0.0034	0.0246	0.0036
	PC2	0.0139	0.0161	0.3234	0.1249	0.0072	0.0952	0.2475	0.2306	0.2013	0.0017
